# Physicochemical, Pharmacokinetic and Cytotoxicity of the Compounds Isolated from an Endophyte *Fusarium oxysporum*: In Vitro and In Silico Approaches

**DOI:** 10.3390/toxins14030159

**Published:** 2022-02-23

**Authors:** Nazia Hoque, Farhana Afroz, Farjana Khatun, Satyajit Roy Rony, Choudhury Mahmood Hasan, Md. Sohel Rana, Md. Hossain Sohrab

**Affiliations:** 1 Department of Pharmacy, Faculty of Science & Engineering, East West University, Dhaka 1212, Bangladesh; nzh@ewubd.edu (N.H.); fkh@ewubd.edu (F.K.); 2 Pharmaceutical Sciences Research Division, Bangladesh Council of Scientific and Industrial Research (BCSIR), Dhaka 1205, Bangladesh; farhana@bcsir.gov.bd (F.A.); satyajit_pharm@bcsir.gov.bd (S.R.R.); 3 Department of Pharmacy, Faculty of Biological Sciences, Jahangirnagar University, Savar, Dhaka 1342, Bangladesh; sohelrana.ju@juniv.edu; 4 Bio-Bio-1 Bioinformatics Research Foundation, Dhaka 1000, Bangladesh; 5 Department of Pharmacy, School of Engineering, Science and Technology, Manarat International University, Dhaka 1212, Bangladesh; cmhasan@gmail.com

**Keywords:** *Fusarium oxysporum*, 3*β*,5*α*-dihydroxy-ergosta-7,22-dien-6-one, 3-(*R*)-7-butyl-6,8-dihydroxy-3-pent-11-enylisochroman-1-one, beauvericin, Vero cell lines, in silico

## Abstract

The present study was intended to characterize the secondary metabolites of the endophyte *Fusarium oxysporum* isolated from the plant *Aglaonema hookerianum* Schott. And to investigate the cytotoxic and other pharmacological properties of the isolated compounds as part of the drug discovery and development process. Different chromatographic techniques were adopted to isolate the bioactive compounds that were identified by spectroscopic techniques. The cytotoxic properties of the compounds were assessed in the Vero cell line via the trypan blue method. Moreover, physicochemical, pharmacokinetic, bioactivity and toxicity profiles of the compounds were also investigated through in silico approaches. After careful spectral analysis, the isolated compounds were identified as 3*β*,5*α*-dihydroxy-ergosta-7,22-dien-6-one (**1**), 3*β*,5*α*,9*α*-trihydroxy-ergosta-7,22-dien-6-one (**2**), *p*-hydroxybenzaldehyde (**3**), 3-(*R*)-7-butyl-6,8-dihydroxy-3-pent-11-enylisochroman-1-one (**4**) and beauvericin (**5**). An in vitro study in the Vero cell line revealed that the presence of the compounds reduced the number of cells, as well as the percentage of viable cells, in most cases. An in silico cytotoxic analysis revealed that compounds **1**, **2** and **5** might be explored as cytotoxic agents. Moreover, compounds **3** and **4** were found to be highly mutagenic. The present study suggested that further thorough investigations are necessary to use these molecules as leads for the cytotoxic drug development process.

## 1. Introduction

Most endophytes are symbiotically associated with their host plants and are able to produce bioactive secondary metabolites without causing apparent damage to the plant. These secondary metabolites become an attractive source for therapeutic compounds, which are still a poorly explored field in drug discovery [1]. The literature has established the genus *Fusarium* as a repository of bioactive compounds, including anti-fungal agents [2], antimicrobial agents [3], fungal toxins [4,5] and immunosuppressive agents [6]. More specifically, the genus *Fusarium* could be a potent source for isolating anticancer compounds such as taxol isolated from *Fusarium redolens* [7], camptothecin and podophyllotoxins isolated from *Fusarium solani* [8,9] and vincristine isolated from *Fusarium oxysporum* [10]. Furthermore, the above examples proved that the production of microorganism-based natural drugs can minimize the vulnerability of plant species as sources of natural drugs. *Fusarium oxysporum* has been established as a promising source of numerous bioactive molecules, such as jasmonic acid and 9,10-dihydrojasmonic acid [11], fumonisin (C1–C4) [12], bikaverin [13], fusarinolic acid, beauvericin, cerevesterol [14], sambutoxin [5], fusarin C [15] and many others.

In the context of our investigation on endophytic fungi [16,17,18,19,20], this study aimed to establish the profiles of the bioactive compounds of the ethyl acetate extract of the endophyte *Fusarium oxysporum*, isolated from the plant *Aglaonema hookerianum* Schott., which led to the identification and characterization of five compounds: 3*β*,5*α*-dihydroxy-ergosta-7,22-dien-6-one (**1**), 3*β*,5*α*,9*α*-trihydroxy-ergosta-7,22-dien-6-one (**2**), *p*-hydroxybenzaldehyde (**3**), 3-(*R*)-7-butyl-6,8-dihydroxy-3-pent-11-enylisochroman-1-one (**4**) and beauvericin (**5**). The physicochemical, bioactivity and ADMET profiles of these metabolites are also reported herein through in silico and in vitro approaches.

## 2. Results

### 2.1. Morphological Identification of the Fungal Strain

**Figure 1:**
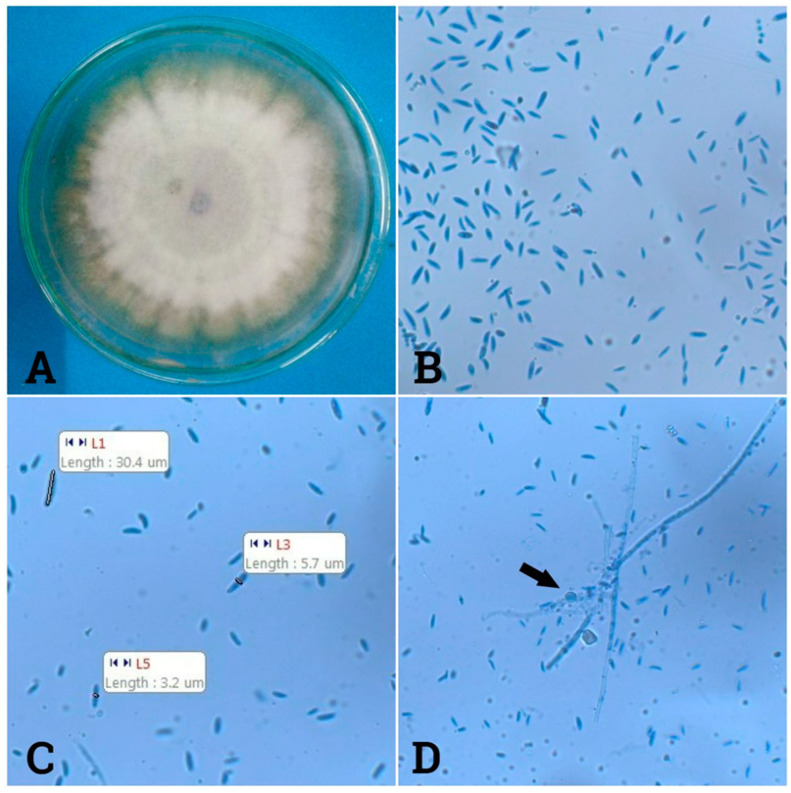
Macroscopic and microscopic views of *Fusarium oxysporum*. (**A**): Colony morphology; (**B**,**C**): Macroconidia and microconidia; (**D**): Chlamydospores indicated by an arrow.

The tested fungal strain was recognized as *Fusarium oxysporum* on the basis of the key features of colony morphology. In this isolate, violet macroconidia were produced in the central spore’s mass and dark magenta or violet pigment were produced in the agar medium. Mycelia was scarce, floccose and white to violet in color (Figure 1). The isolate also produced abundant, pale orange sparsed sporodochia. Most macroconidia were of short to medium length, slightly curved or straight, sometimes with a slight hook, relatively slender and thin-walled and usually 3-septate. The microconidia were elliptical, oval or reniform (kidney shaped) and usually non-septated. The chlamydospores were infinite singly or in pairs and quickly formed within 2 to 4 weeks for most isolates [21].

### 2.2. Characterization of Compound ***1*** as 3β,5α-Dihydroxy-ergosta-7,22-diene-6-one

**Figure 2:**
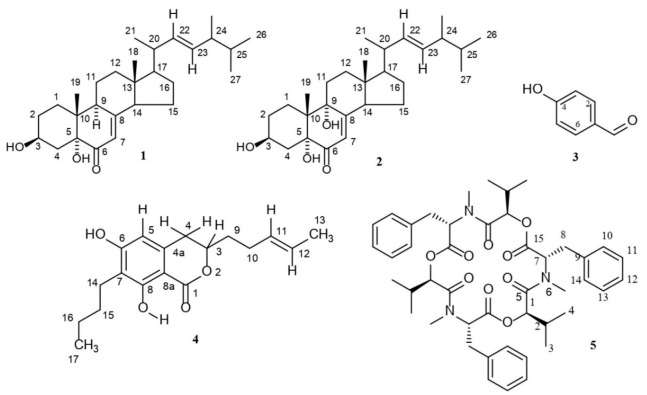
The structures of compounds (**1**–**5**) isolated from *Fusarium oxysporum*.

In the ^1^H nuclear magnetic resonance (NMR) spectrum, the existence of one one-proton multiplet at *δ* = 4.07 ppm and one one-proton singlet at *δ* = 5.67 ppm represents H-3 and H-7 of a sterol molecule. The two double-doubles at *δ* = 5.18 ppm and *δ* = 5.26 ppm could be assigned to two olefinic protons located at the side chain of steroidal compounds. The ^1^H NMR data also revealed that compound **1** (Figure 2) contains six methyl signals at *δ* = 1.05, 0.97, 0.93, 0.84, 0.87 and 0.62 ppm. Two signals at *δ* = 67.5 and 71.1 in the ^13^C NMR spectrum clearly specify the presence of two hydroxyl groups attached to the C-3 and C-5 positions and a signal at *δ* = 197.4 showed the presence of a carbonyl group at the C-6 position (Figures S3–S8). Finally, based on the above analysis, compound **1** was identified as 3*β*,5*α*-dihydroxy-ergosta-7,22-diene-6-one, a steroidal molecule containing two hydroxyl groups, two double bonds, six methyl groups and a carbonyl group with 28 carbons [22].

### 2.3. Characterization of Compound ***2*** as 3β,5α,9α-Trihydroxy-ergosta-7,22-diene-6-one

The structure of compound **2** (Figure 2) was elucidated by a comparison of its spectral data with those of compound **1**. Differences were found in the C-9 substituent. Although both the ^1^H NMR and ^13^C NMR spectra of compound **2** were in close correspondence to those of compound **1**, one new signal at *δ* = 74.7 appeared, and the signal at *δ* = 44.7 disappeared in the ^13^C NMR spectrum of compound **2** (Figures S9–S14). The high resolution electrospray ionization mass spectrometry (HRESIMS) spectrum with [M + Na]^+^ at *m/z* = 467.3142, in conjunction with the other spectral data, suggested the molecular formula C_28_H_44_O_4_ (Figure S15), and the compound was recognized as 3*β*,5*α*,9*α*-trihydroxy-ergosta-7,22-diene-6-one [23].

### 2.4. Characterization of Compound ***3*** as p-Hydroxybenzaldehyde

The ^1^H NMR and ^13^C NMR spectra of compound **3** (Figure 2) showed signals for an aldehydic proton at *δ* = 9.76 and *δ* = 191.4, respectively. The proton signals at *δ* = 6.89 and 7.73 represent the presence of disubstituted aromatic protons, which is also supported by the presence of olefin methine carbon signals at *δ* = 132.4 and 115.8 and one oxygenated olefin carbon signal at *δ* = 163.2. A comparison of these data with the published values [24] allowed for the characterization of this compound as *p*-hydroxybenzaldehyde (Figures S16–S19).

### 2.5. Characterization of Compound ***4*** as 3-(R)-7-Butyl-6,8-dihydroxy-3-pent-11-enylisochroman-1-one

The presence of one one-proton singlet at *δ* 6.21 in the ^1^H NMR spectrum is attributed to one aromatic proton. The presence of only one aromatic proton indicated that compound **4** (Figure 2) might carry a pentasubstituted benzene ring. The presence of one sharp one-proton singlet at *δ* = 11.44 could be ascribed to one phenolic chelated hydroxyl group and the doublet signal at *δ* = 1.66 (*J* = 5.6 Hz) could be ascribed for the methyl group at C-13. Two double doublets at *δ* = 5.51 and 5.43 confirm the presence of a trans-olefinic bond between C-11 and C-12. The signal at *δ* = 170.5 in the ^13^C NMR spectrum revealed the presence of a carbonyl carbon in lactone moiety and an oxygenated methine carbon at C-3 (*δ* = 78.5). The ^13^C NMR spectrum also showed two signals at *δ* = 160.0 and 162.2 for the two phenolic carbons at C-6 and C-8, respectively. The ^13^C and DEPT-135 NMR spectra revealed the presence of two methyls, four methines, six methylenes and six quaternary carbons in compound **4** (Figures S20–S28). An accurate mass measurement of compound **4** obtained by HRESIMS yielded a parent mass of *m/z* 327.1565 in positive ionization mode, corresponding to the sodium adduct [M + Na]^+^ with a molecular formula of C_18_H_24_O_4_ (Figure S29). On the basis of the above observations and compared with the literature data [25], compound **4** was identified as 3-(*R*)-7-butyl-6,8-dihydroxy-3-pent-11-enylisochroman-1-one, a dihydroisocoumarin derivative.

### 2.6. Characterization of Compound ***5*** as Beauvericin

The resonance at *δ* = 0.41, 0.82 and 3.04 in the ^1^H NMR and at *δ* = 17.4, 18.3 and 32.2 in the ^13^C NMR spectra, in conjunction with the DEPT-135 spectrum, could be attributed to three methyl groups in compound **5** (Figure 2). In a similar way, *δ*_H_ = 2.97 and *δ*_C_ = 34.7 represented one methylene, *δ*_H_ = 1.99, 4.88 and 5.58 along with *δ*_C_ = 29.7, 75.6 and 57.2 represented three methines and *δ*_H_ = 7.24 and *δ*_C_ = 126.8–128.8 could be attributed to a benzene ring. Furthermore, the signals at *δ*_H_ = 3.04 and *δ*_C_ = 32.2 revealed the presence of a N-methyl amino acid moiety of beauvericin (Figures S30–S37). The ^13^C NMR spectrum confirms the presence of fifteen carbons, and an X-ray crystallography report (Figure S39) confirms three moieties with symmetrical structures in the compound. The HRESIMS spectra of compound **5** yielded a parent mass of *m/z* 806.3984, corresponding to the sodium adduct [M + Na]^+^ with a molecular formula of C_45_H_57_N_3_O_9_ (calcd. mass 806.9404 [C_45_H_57_N_3_O_9_ + Na]^+^), accounting for 19 degrees of unsaturation (Figure S38). By careful inspection of its ^1^H and ^13^C NMR spectral data along with mass and X-ray crystallography reports [26], this compound was characterized as beauvericin.

### 2.7. Trypan Blue Test

**Figure 3:**
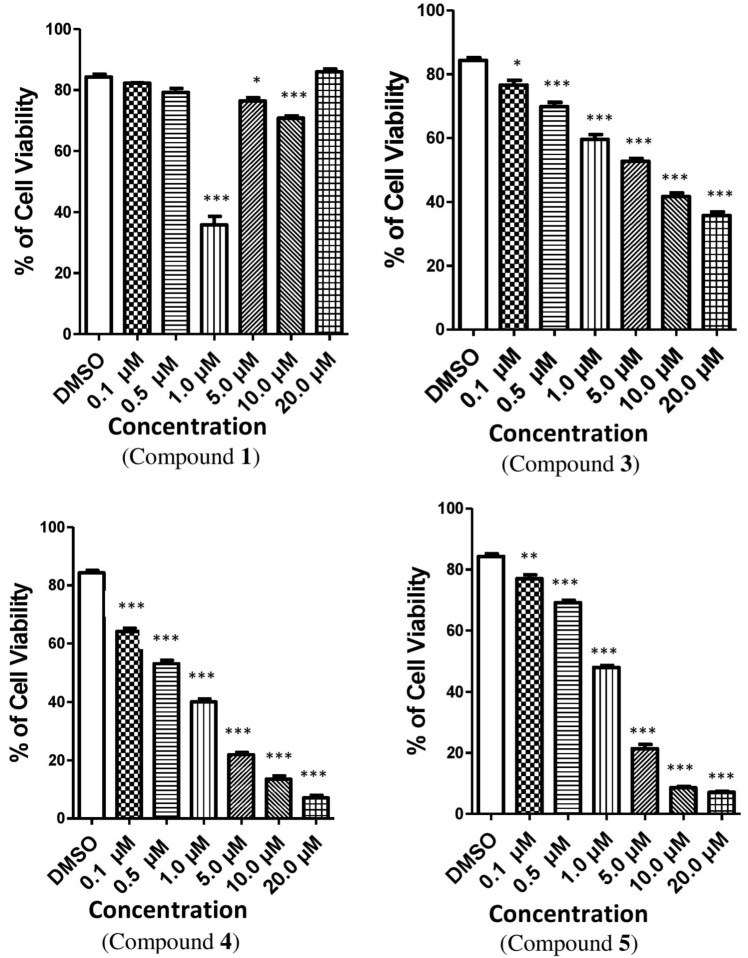
The effect of compounds **1**, **3**, **4** and **5** on the percentage of cell viability of Vero cells. The relative fold changed the cell viability in Vero cells treated with these compounds when compared with the vehicle (DMSO). Here, compound **1** = 3*β*,5*α*-dihydroxy-ergosta-7,22-diene-6-one, compound **3** = *p*-hydroxybenzaldehyde, compound **4** = 3-(*R*)-7-butyl-6,8-dihydroxy-3-pent-11-enylisochroman-1-one, compound **5** = Beauvericin. Different shaded bars represent different concentrations of the test samples and the vehicle DMSO. The results are expressed as the mean ± SEM (*n* = 3). The degrees of significance calculated by employing ANOVA with Post Hoc Tukey’s test were * *p* < 0.05, ** *p* < 0.01 and *** *p* < 0.001.

**Figure 4:**
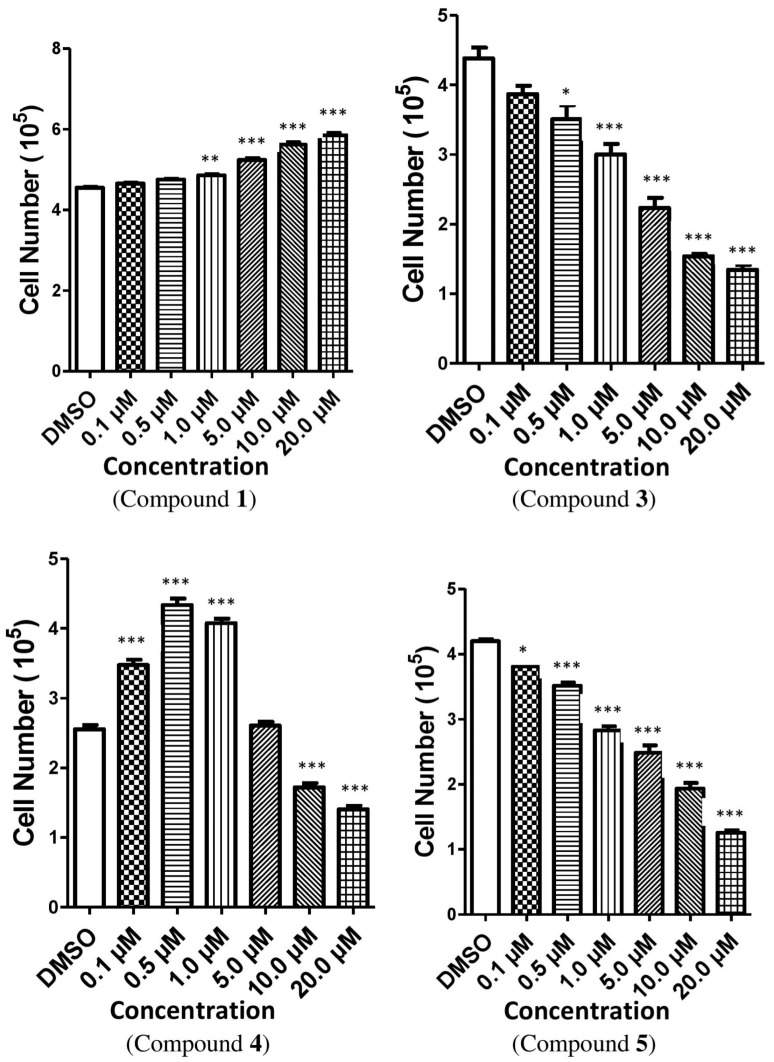
The effect of compounds **1**, **3**, **4** and **5** on the total cell number of Vero cells. The relative fold changed in the total cell number of Vero cells treated with these compounds when compared with the vehicle (DMSO). Here, compound **1** = 3*β*,5*α*-dihydroxy-ergosta-7,22-diene-6-one, compound **3** = *p*-hydroxybenzaldehyde, compound **4** = 3-(*R*)-7-butyl-6,8-dihydroxy-3-pent-11-enylisochroman-1-one, compound **5** = Beauvericin. Different shaded bars represent different concentrations of the test samples and the vehicle DMSO. The results are expressed as the mean ± SEM (*n* = 3). The degrees of significance calculated by employing ANOVA with Post Hoc Tukey’s test were * *p* < 0.05, ** *p* < 0.01 and *** *p* < 0.001.

**Figure 5:**
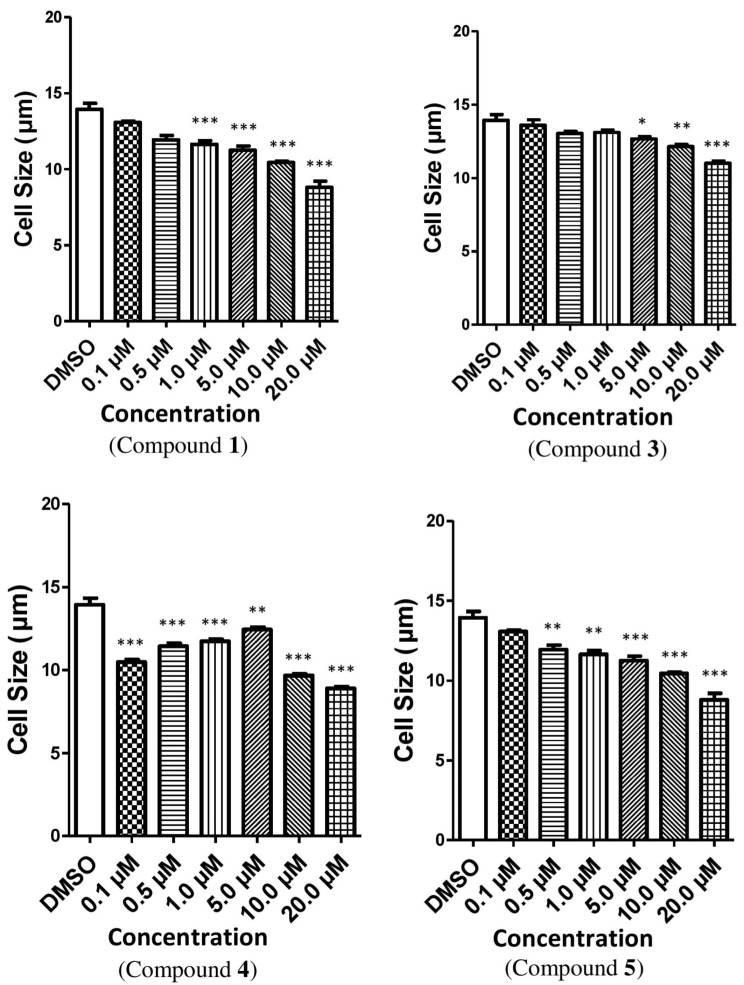
The effect of compounds **1**, **3**, **4** and **5** on the cell size of Vero cells. The relative fold changed in the cell size of Vero cells treated with these compounds when compared with the vehicle (DMSO). Here, compound **1** = 3*β*,5*α*-dihydroxy-ergosta-7,22-diene-6-one, compound **3** = *p*-hydroxybenzaldehyde, compound **4** = 3-(*R*)-7-butyl-6,8-dihydroxy-3-pent-11-enylisochroman-1-one, compound **5** = Beauvericin. Different shaded bars represent different concentrations of the test samples and the vehicle DMSO. The results are expressed as the mean ± SEM (*n* = 3). The degrees of significance calculated by employing ANOVA with Post Hoc Tukey’s test were * *p* < 0.05, ** *p* < 0.01 and *** *p* < 0.001.

The cytotoxic efficacy of the isolated compounds was assessed against the Vero cell line (African Green Monkey kidney cell) after 24 h of exposure. The toxicity of the compounds was determined where a dose-dependent reduction in cell viability was observed. In case of compound **1**, the percentage of viable cells was lowered significantly at the concentration of 1.0 µM, but afterwards the percentage of viable cells was augmented with the increased concentrations (Figure 3). On the other hand, compounds **3**, **4** and **5** reduced the cell viability with their increased concentrations (Figure S40). The results were consistent for the total cell number for all the compounds except compound **1**, where total cell growth was induced with the increased concentrations (Figure 4). On the other hand, cell size was reduced with the higher concentrations of all the tested compounds except for compound **4**. In case of compound **4**, cell size was enlarged and leveled off when the concentration was 5.0 µM; afterwards, cell size dropped with the augmented concentrations of the compound (Figure 5). It is necessary to conduct further specific and higher studies to explore the mechanism of anticancer efficacy of these compounds.

### 2.8. In Silico Prediction of Physicochemical and Pharmacological Properties

In silico analyses are often performed to predict important information such as physicochemical, pharmacodynamic and pharmacokinetic properties of bioactive compounds in a faster manner. It has become the method of choice as an early drug discovery process to improve the efficacy and druggability properties, as well as to avoid various toxicities and other side effects of drug candidates. Therefore, the probability of success in drug development has increased and overall expenses have decreased [27]. In this study, several online tools were used to assess the physicochemical, bioactivity, absorption, distribution, metabolism, elimination and toxicity (ADMET) properties of the isolated compounds (Table 1).

### 2.9. Physicochemical Properties

Molecular descriptors such as lipophilicity (n-octanol/water partition coefficient, Log P_O/W_), solubility (Log S), hydrogen bond donor and acceptor, rotational bonds, topological polar surface area (TPSA) and molar refractivity contribute to the potency, selectivity against the target and ADMET profiles of the drug candidates [27]. Therefore, this study predicted the physicochemical properties of isolated molecules using the SwissADME tool (http://www.swissadme.ch/ by the Swiss Institute of Bioinformatics, Switzerland) (accessed on 10 November 2021) (Table 1). As stated in Lipinski’s rule of five, the Log P_O/W_ of a compound needs to be less than 5.0 for adequate absorption through the cell membrane [28]. The consensus Log P_O/W_ value obtained from five different predictions (iLogP, WLogP, XLogP3, Silicos-IT and MLogP) indicated that compounds **2**, **3** and **4** met this criterion, whereas compounds **1** and **5** have slightly higher values than 5.0. The water solubility (Log S) of the isolated compounds was also determined using three different predictions (ESOL, Ali and Slilicos-IT), where the order of the solubility was compound **3** > **2** > **1** and **4** > **5**. To determine the probability of the molecules to be active orally, drug-likeness was assessed through the Lipinski, Veber, Ghose, Muegge and Egan filters (Table 1), which were developed on the basis of several physicochemical features. It is a qualitative hypothesis to delineate the relationship between physicochemical and pharmacokinetic parameters [29]. In this study, compound **4** satisfied all the rules of five different filters except for one rule (XLogP3 > 5) of Muegge’s filter, and compound **2** violated only two rules of Ghose’s filter. Compounds **1** and **3** met the parameters of Lipinski, Veber and Egan’s filters, but failed to satisfy all the parameters of Ghose and Muegge’s filters. Compound 5 met only the rules of Veber’s filter.

### 2.10. Bioactivity

The Molinspiration online tool (https://www.molinspiration.com/ by Molinspiration Cheminformatics, Slovak Republic) (accessed on 11 November 2021) provides an activity score of the compounds between −3.0 and 3.0 to identify their activity as G-protein coupled receptor (GPCR) ligands, nuclear receptor ligands, protease inhibitors, ion channel modulators, kinase inhibitors and enzyme inhibitors. Molecules with the highest score possess the highest possibility to be active. Here, compounds **1**, **2**, and **4** were found to be biologically active as nuclear receptor ligands, where compound **2** (0.91) has the highest bioactivity score, followed by compound **1** (0.75) and compound **4** (0.49). These compounds also have similar bioactivity scores to act as enzyme inhibitors (Table 1). On the contrary, compounds **3** and **5** showed bioactivity scores <0.00, which indicates their inactivity against these targeted sites.

### 2.11. Pharmacokinetic Properties

Pharmacokinetic features such as the absorption, distribution, metabolism and excretion of drug molecules play a major role in reaching the clinic for end users. An analysis of the isolated compounds using the SwissADME tool revealed that all compounds except for compound **5** were expected to be highly absorbable through the gastrointestinal (GI) tract. Compounds **3** and **4** showed their ability to be permeable to the blood brain barrier (BBB), whereas compounds **1**, **2** and **5** were non-permeable to the BBB. In case of skin permeability, the higher the negative log Kp value, the lower the skin permeation of the molecule [29]. Therefore, the permeability of the compounds to the stratum corneum of the skin is as follows: compound **4** > **1** > **5** > **2** > **3**. P-glycoprotein (P-gp), a protein of intestinal cell membrane, has the capability to pump out the absorbed drug into the gut lumen. Compounds **2** and **5** were predicted to be P-gp substrate, whereas compounds **1**, **3** and **4** were non-substrate to this membranous protein. An important class of enzymes for metabolism is Cytochrome P_450_, and inhibition or induction of these enzymes by drug molecules produces undesirable metabolites, which may result many unwanted drug reactions [30]. In this study, all compounds except for compound **4** were found to be non-inhibitors of important isoenzymes such as CYP2C19, CYP1A2, CYP2C9, CYP3A4 and CYP2D6. Compound **4** was predicted to be an inhibitor of the CYP1A2 and CYP2C9 isoenzymes (Table 1).

### 2.12. In Silico Analysis of Toxic Properties

An analysis of the compounds by the Osiris Property Explorer revealed that compounds **1**, **2** and **5** were predicted to be safe, as toxicological parameters such as mutagenicity, tumorigenicity, irritation and effect on the reproductive system were absent. Mutagenic and irritant effects were highly present in compound **3**, whereas compound **4** has moderate mutagenic properties. The maximum tolerated dose (MTD) of the compounds for human use was predicted through the pkCSM online tool (http://biosig.unimelb.edu.au/pkcsm/ by Bio21 Institute, University of Melbourne, Melbourne, Australia) (accessed on 7 November 2021), where compounds having a value below 0.477 (log (mg/kg/day)) needed to be given in low doses due to their toxic effects. The MTD of isolated compounds **1**, **2**, **4** and **5** was found to be low, indicating their capacity to act as toxic, whereas compound **3** can be given at high doses due to its lower potency of toxicity. The activity of the compounds against human ether-a-go-go related gene (hERG) was also investigated. Potassium channels are encoded by two genes (hERG I and hERG II), and inhibition of these channels develops an acquired long QT syndrome that may lead to severe ventricular arrhythmia. An in silico prediction against hERG I revealed no inhibitory features of the isolated compounds. Compounds **1**, **2** and **3** were not found as inhibitors against hERG II, whereas compounds **4** and **5** showed their probability to act as inhibitors for hERG II. Drug-induced liver toxicity is an important parameter of drug development. Using the pkCSM prediction tool, compounds **1**, **2** and **5** were found as hepatotoxic, whereas compounds **3** and **4** did not show any relation to hepatotoxicity (Table 1).

### 2.13. In Silico Cell Line Toxicity

The in silico cytotoxic activity of the isolated compounds in different cell lines is shown in Table 2, where three cancerous cell lines with a high probability value are represented for each compound. In CLC-Pred (http://www.way2drug.com/cell-line/ developed by Institute of Biomedical Chemistry of Russian Academy of Medical Sciences, Moscow, Russia) (accessed on 15 November 2021), the activity of the compounds was calculated based on the statistics of the Multilevel Neighbourhood of Atoms (MNA) descriptors and labelled with the probability of the compound being active (Pa) or inactive (Pi), ranging from 0.000 to 1.000 [31].

An investigation through CLC-Pred revealed that compound **1** showed an almost similar probability of being active against colon adenocarcinoma (HCC 2998), ovarian adenocarcinoma (OVAR-5) and lung carcinoma (DMS-114), with values of 0.633, 0.616 and 0.610, respectively. Compound **2** showed a probability of being active against lung carcinoma (DMS-114) and small cell lung carcinoma (NCI-H187) with values of 0.584 and 0.501, respectively. However, it also possessed the probability (0.413) to be cytotoxic for normal cell line embryonic lung fibroblast (WI-38 VA13). Compound **3** demonstrated a higher probability (0.664) of being active against the oligodendroglioma (Hs 683) cell line rather than small cell lung carcinoma (NCI-H187) and non-small cell lung carcinoma (HOP-18), which possessed probabilities of 0.594 and 0.431, respectively. Compound **4** showed its prominence of cytotoxicity in a similar trend against melanoma (A2058), small cell lung carcinoma (NCI-H187) and lung carcinoma (DMS-114) with probabilities of 0.499, 0.488 and 0.448, respectively. The prediction of cytotoxicity of compound **5** was found to be higher against glioblastoma (SF-268) than breast adenocarcinoma (MDA-MB-231) and pancreatic carcinoma (MIA PaCa-2).

## 3. Discussion

Nature is considered to be the best source to identify new bioactive compounds with potent anticancer activity. More than 60% of clinically used drugs against cancer are developed from natural sources, and they demonstrate their activity via apoptosis, autophagy, immune function regulation or cell proliferation inhibition [32]. Therefore, the focus of this study was to investigate the toxicity profiles of five compounds obtained from *Fusarium oxysporum* isolated from the plant *Aglaonema hookerianum* Schott. Moreover, the physicochemical, bioactivity and pharmacokinetic profiles of these molecules were analyzed to determine their eligibility to be the drug/lead molecules for the anticancer drug development process.

Compounds *p*-hydroxybenzaldehyde (**3**), 3-(*R*)-7-butyl-6,8-dihydroxy-3-pent-11-enylisochroman-1-one (**4**) and beauvericin (**5**) inhibited total cells in number and viable cells in percentage in transformed Vero cells. Similar to our observation, various laboratories also found that compound **5** inhibited different transformed cell lines, such as Vero cells, human breast cancer cells BC-1, human monocytic lymphoma cells U-937, etc. [33,34,35,36,37,38]. Previous in vitro studies have reported that compound **5** has slowed the proliferation of different types of cells. This inhibition was also dependent on dose and time of incubation [39,40]. The trypan blue results suggested that compounds **3**, **4** and **5** may hinder cell growth or initiate apoptosis or necrosis. It is also indicated that compounds **3**, **4** and **5** induced cell damage of some sort, but it is not known whether this is apoptosis or necrosis. Apoptosis or necrosis induced by extracellular signaling pathways in in vitro cultures would be a possible explanation of the reduction of the viability of cells in culture. It is not well established from our results that there was any co-relation between the inhibition of cell proliferation, cell size and total cell number, but it is presumed that any co-relation may remain. Compound 3*β*,5*α*-dihydroxy-ergosta-7,22-diene-6-one (**1**) decreased the percentage of viable Vero cells at a certain concentration, but afterwards it was augmented. Compound **1** also increased the total cell number, opposite to the actions of the other compounds. This finding revealed that compound **1** may activate cell division or inhibit apoptosis or necrosis in Vero cells. This result indicated that compound **1** may protect cells, especially kidney cells, from cell damage, though further thorough investigation is required to confirm this.

The physicochemical, bioactivity, pharmacokinetic and toxic properties of the isolated compounds were evaluated through different online tools. Compound **4** met almost all the eligibility criteria of different drug-likeness filters and possessed the probability to be a nuclear receptor ligand. It showed less probability to be active against different cancer cell lines. However, it demonstrated the probability to have moderate mutagenic properties and to be an inhibitor of CYP1A2 and CYP2C9 isoenzymes and hERG II. Compound **2** satisfied the criteria of all drug-likeness features except for the Ghose filter. It has shown the highest probability (0.91) of being a nuclear receptor ligand. Moreover, it may be a P-gp substrate and a non-inhibitor of important isoenzymes. However, it showed a probability for hepatotoxicity along with activity in both cancerous and non-cancerous cell lines. Compounds **1** and **3** met the drug-likeness criteria of Lipinski, Veber and Egan’s filters. Compound **1** showed its probability to be a nuclear receptor ligand, whereas compound **3** is predicted to be a blood brain barrier (BBB)-permeant molecule and demonstrated negative bioactivity scores against all target sites. Compound **1** showed the probability to be active against colon and ovarian adenocarcinoma cells and compound **4** demonstrated its activity against Oligodendroglioma (Hs 683). Moreover, compound **1** did not show any undesired effects except for hepatotoxicity, while compound **3** demonstrated a probability to be highly mutagenic and highly irritant. Compounds **1** and **2** have almost similar structures with the only exception at C9 position, where compound **2** possesses a hydroxyl moiety. The presence of this functional group made an impact on the solubility and absorption (being a P-gp substrate) profiles (Table 1) of compound **2**. Compound **5** is lipophilic in nature and met only the drug-likeness features of Veber’s filter. It showed a higher probability of being active against glioblastoma (SF-268), breast adenocarcinoma (MDA-MB-231) and pancreatic carcinoma (MIA PaCa-2) cell lines (Table 2), which is supported by an in vitro study conducted by Zhan et al. [35]. It was found to be an inactive molecule (bioactivity score <0.00) against the target sites available in the Molinspiration tool, which may be due to its high molecular weight. A recent in vitro study has reported it as a potent anticancer molecule against KB cells through the inhibition of acetyl-CoA acetyltransferase 1 (ACAT1) [41]. However, this compound requires structural modification to improve oral bioavailability as well as to avoid the features of hERG II inhibitor and hepatotoxicity.

## 4. Conclusions

Our present study highlighted the possibility of establishing *Fusarium oxysporum* as a treasury of promising secondary metabolites. Considering in vitro and in silico analyses, it can be summarized that the isolated compounds can be used as prominent lead compounds for anticancer drug development, and further detailed studies are required to design the synthetic analogs of these compounds along with the establishment and optimization of their therapeutic and pharmacokinetic activities.

## 5. Materials and Methods

### 5.1. Collection and Identification of the Plant Material

In August 2014, the plant *A. hookerianum* was collected from Pablakhali, Chittagong Hill Tracts, Bangladesh. The plant’s taxonomical characterization was completed by the Bangladesh National Herbarium and a specimen bearing the no. DACB 40633 was kept for future reference (Figure S1).

### 5.2. General Experimental Procedures

The silica gel for column chromatography and the silica plates for thin layer chromatography were procured from Merck, Darmstadt, Germany. A Bruker 400 MHz spectrophotometer was operated to record all the NMR spectra of the compounds using deuterated solvents (CDCl_3_ and MeOD). The Vero cell lines were supplied by CLS cell lines service GmbH, 605372, Eppelheim, Germany.

### 5.3. Isolation and Extraction of Fungal Material

After surface sterilization, the edges of sliced leaves, roots and petioles of *A. hookerianum* were cut under sterile conditions and placed on a water agar media. To inhibit bacterial growth, streptomycin with a concentration of 100 mg L^−1^ was mixed with the media. Within 4–5 weeks, fungal growth initiated from the sliced segments of the plant that were taken off of the water agar media and placed onto a potato dextrose agar (PDA) medium [16,17,18,19,20]. Two endophytic fungi were collected from the petiole and characterized as *Fusarium* sp. based on their macroscopic and microscopic morphological characters. Similarly, two endophytic fungi were collected from the leaf and characterized as *Colletotrichum* sp. One of the *Fusarium* sp. was selected to isolate bioactive compounds based on its preliminary bioassay screening and was subjected to elaborated microscopical identification to confirm the species (Figure S2). The selected fungal strain was cultivated at a large scale by using approximately 20 L of PDA media and maintaining the temperature at 28 ± 2 °C. After 21 days, the cultured media with the matured fungal materials were soaked with ethyl acetate for 7 days with intermittent shaking. After that period, the solvent was filtered sequentially through cotton plug and filter paper. The collected filtrate was concentrated to obtain the crude extract (8.0 g).

### 5.4. Identification of the Selected Endophytic Fungus

A fungal isolate was cultured on PDA for 7 days for morphological examination. The isolate was characterized by its macroscopic and microscopic characteristics (i.e., color, size, length and width of macroconidia and microconidia and septation) according to the manual [21].

### 5.5. Isolation of Compounds

The fungal extract was loaded into a silica gel column using different concentrations of petroleum ether-EtOAc-MeOH as mobile phase and yielded 15 fractions (FS-1 to FS-15). Compound **1** (8.0 mg, brown solid) was obtained from FS-10 (petroleum ether/70% EtOAc), compound **3** (9.50 mg, white crystal) was obtained from FS-7 (petroleum ether/40% EtOAc) and compound **4** (12.40 mg, white powder) was obtained from FS-6 (petroleum ether/30% EtOAc) by solvent treatments of varying polarity. The column fraction FS-9 (petroleum ether/60% EtOAc) was subjected to preparative thin layer chromatography using toluene/40% EtOAc as a mobile phase (2 developments) to obtain compound **2** (3.20 mg, white powder). Column fraction FS-11 (petroleum ether/90% EtOAc) was subjected to column chromatography using *n*-hexane-CH_2_Cl_2_-MeOH as a mobile phase to yield 8 fractions (FSS-1 to FSS-8). Compound **5** (21.0 mg, needle shaped white crystal) was obtained from the subfraction FSS-6 eluted with CH_2_Cl_2_/1% MeOH.

### 5.6. Compound ***1*** (3β,5α-Dihydroxy-ergosta-7,22-dien-6-one)

Brown solid; ^1^H NMR (400 MHz, CDCl_3_): *δ* 5.67 (2H, s, H-7), 5.26 (1H, dd, *J* = 15.2, 7.6 Hz, H-23), 5.18 (1H, dd, *J* = 8.0, 15.2 Hz, H-22), 4.07 (1H, m, H-3), 1.05 (3H, d, *J* = 6.8 Hz, H-21), 0.97 (3H, s, H-19), 0.93 (3H, d, *J* = 6.8 Hz, H-28), 0.87 (3H, d, *J* = 5.6 Hz, H-27), 0.84 (3H, d, *J* = 6.4 Hz, H-26), 0.62 (3H, s, H-18). ^13^C NMR (100 MHz, CDCl_3_): *δ* 197.4 (C-6), 152.0 (C-8), 135.0 (C-22), 132.5 (C-23), 119.7 (C-7), 71.1 (C-5), 67.5 (C-3), 56.1 (C-17), 48.3 (C-14), 44.7 (C-9), 43.9 (C-13), 42.8 (C-24), 40.5 (C-10), 40.2 (C-20), 36.4 (C-4), 33.7 (C-12), 33.0 (C-25), 30.2 (C-2), 28.6 (C-11), 27.6 (C-16), 24.5 (C-1), 21.9 (C-21), 21.8 (C-15), 21.1 (C-19), 19.9 (C-26), 19.6 (C-27), 17.5 (C-28), 12.7 (C-18).

### 5.7. Compound ***2*** (3β,5α,9α-Dihydroxy-ergosta-7,22-dien-6-one)

White powder; ESI-MS: [M + Na]^+^ *m/z* 467.3142 (calcd for C_28_H_44_O_4_Na, 467.6374). ^1^H NMR (400 MHz, CDCl_3_): *δ* 5.69 (2H, d, *J* = 1.6 Hz, H-7), 5.26 (1H, dd, *J* = 7.2, 15.2 Hz, H-23), 5.18 (1H, dd, *J* = 8.0, 15.4 Hz, H-22), 4.07 (1H, m, H-3), 1.06 (3H, s, H-21), 1.04 (3H, s, H-19), 0.94 (3H, d, *J* = 6.8 Hz, H-28), 0.85 (3H, t, *J* = 6.8 Hz, H-26), 0.85 (3H, t, *J* = 6.8 Hz, H-27), 0.64 (3H, s, H-18). ^13^C NMR (100 MHz, CDCl_3_): *δ* 197.4 (C-6), 164.2 (C-8), 135.0 (C-22), 132.5 (C-23), 119.9 (C-7), 79.8 (C-5), 74.7 (C-9), 67.2 (C-3), 56.0 (C-17), 51.7 (C-14), 45.3 (C-13), 42.8 (C-24), 41.8 (C-10), 40.2 (C-20), 37.2 (C-4), 34.9 (C-12), 33.0 (C-25), 30.1 (C-2), 28.8 (C-11), 27.8 (C-16), 25.4 (C-1), 22.4 (C-15), 21.0 (C-21), 20.4 (C-26), 20.3 (C-19), 19.9 (C-27), 17.6 (C-28), 12.2 (C-18).

### 5.8. Compound ***3*** (p-Hydroxybenzaldehyde)

White crystal; ^1^H NMR (400 MHz, CDCl_3_): *δ* 9.76 (1H, s, -CHO), 7.73 (2H, d, *J* = 8.6 Hz, H-2, H-6), 6.89 (2H, d, *J* = 8.6 Hz, H-3, H-5). ^13^C NMR (100 MHz, CDCl_3_): *δ* 191.4 (-CHO), 163.2 (C-4), 132.4 (C-2, C-6), 128.8 (C-1), 115.8 (C-3, C-5).

### 5.9. Compound ***4*** (3-(R)-7-Butyl-6,8-dihydroxy-3-pent-11-enylisochroman-1-one)

White powder; ESI-MS: [M + Na]^+^ *m/z* 327.1565 (calcd for C_18_H_24_O_4_Na, 327.3712). ^1^H NMR (400 MHz, CDCl_3_): *δ* 11.44 (s, OH-18), 6.21 (1H, s, H-5), 5.51 (1H, dd, *J* = 7.2, 14.8 Hz, H-12), 5.43 (1H, dd, *J* = 6.0 15.2 Hz, H-11), 4.53 (1H, m, H-3), 2.84 (2H, m, H-4), 2.65 (2H, t, *J* = 7.6 Hz, H-14), 2.21 (2H, m, H-10), 1.94 (2H, m, H-9), 1.66 (3H, d, *J* = 5.6 Hz, H-13), 1.54 (2H, m, H-15), 1.42 (2H, m, H-16), 0.96 (3H, t, *J* = 7.2 Hz, H-17). ^13^C NMR (100 MHz, CDCl_3_): *δ* 170.5 (C-1), 162.2 (C-8), 160.0 (C-6), 138.2 (C-4a), 129.5 (C-11), 126.4 (C-12), 114.7 (C-7), 105.9 (C-5), 101.6 (C-8a), 78.5 (C-3), 34.5 (C-9), 32.9 (C-4), 30.9 (C-15), 27.7 (C-10), 22.8 (C-16), 22.3 (C-14), 17.9 (C-13), 13.9 (C-17).

### 5.10. Compound ***5*** (Beauvericin)

Needle shaped white crystal; ESI-MS: [M + Na]^+^ *m/z* 806.3984 (calcd for C_45_H_57_N_3_O_9_Na, 806.9404). ^1^H NMR (400 MHz, CDCl_3_): *δ* 7.24 (5H, m, H-10, H-11, H-12, H13, H-14), 5.58 (1H, d, *J* = 7.8 Hz, H-7), 4.88 (1H, d, *J* = 8.4 Hz, H-1), 3.4 (1H, dd, *J* = 4.6, 14.6 Hz, CH(H)-8b), 3.04 (3H, s, N-CH_3_), 2.97 (1H, dd, *J* = 12.2, 14.4 Hz, H-8a), 1.99 (1H, m, H-2), 0.82 (3H, d, *J* = 6.8 Hz, CH_3_-3), 0.41 (3H, d, *J* = 6.8 Hz, CH_3_-4). ^13^C NMR (100 MHz, CDCl_3_): *δ* 169.9 (CO-5, CO-15), 136.5 (C-9), 128.8 (C-10, C-14), 128.6 (C-11, C-13), 126.8 (C-12), 75.6 (C-1), 57.2 (C-7), 34.7 (C-8), 32.2 (N-CH_3_), 29.7 (C-2), 18.3 (CH_3_-3), 17.4 (CH_3_-4).

### 5.11. In Vitro Cytotoxicity Analysis by Trypan Blue Assay

The investigation of cytotoxicity of the isolated compounds was conducted using a modified trypan blue exclusion method [42,43]. The Vero cell line was used to evaluate the cytotoxicity and was cultured at 37 °C following the process narrated by Khan et al., 2018 [17]. Different doses (0.1 to 20 μg/mL) of the compounds were added into T-25 cell culture containers containing around 2.5 × 10^6^ cells and incubated for 24 h. After the incubation period, the treated cells were collected using 0.5% trypsin. Trypan blue (0.4% *w/v*) was used to make the unviable cells stained and viable cells remained unstained. An automated cell counter was used to count the number of unviable (stained) cells [44]. The percentage of unviable cells was calculated as follows:

Percentage of unviable cells = [number of unviable cells/total number of cells] × 100.

### 5.12. In Silico Analysis of Physicochemical, Bioactivity, Pharmacokinetic and Toxicity Properties

Several online tools were used to assess the physicochemical, bioactivity and ADMET properties of the isolated compounds. The physicochemical, pharmacokinetic and medicinal chemistry aspects of the compounds were screened through the SwissADME tool (http://www.swissadme.ch/index.php) (accessed on 10 November 2021). The Molinspiration online tool version 2018.03 (https://www.molinspiration.com/) (accessed on 11 November 2021) was used to assess the bioactivity score of the compounds against drug targets such as GPCR, kinase enzymes, ion channels, nuclear receptors and other enzymes. The toxicity risks such as the mutagenic, tumorigenic, irritant and reproductive effects of the compounds were determined using the Osiris Property Explorer, and other toxic features were analyzed via the pkCSM online tool (http://biosig.unimelb.edu.au/pkcsm/) (accessed on 7 November 2021). In each case, canonical simplified molecular input line entry system (SMILES) notations of the isolated compounds were fed into the tools to obtain the outcomes.

### 5.13. In Silico Cell Line Toxicity Analysis

The cytotoxic effect of the compounds was screened in both normal (non-tumor) and cancer cell lines by a web service (http://way2drug.com/Cell-line/) (accessed on 15 November 2021) known as the Cell Line Cytotoxicity Predictor (CLC-Pred), where the cytotoxicity of chemicals is predicted based on a PASS (Prediction of Activity Spectra for Substances) algorithm using a training dataset of 59,882 cytotoxic compounds obtained from the experimental data of ChEMBL (version 23) (https://www.ebi.ac.uk/chembldb/) (accessed on 15 November 2021) to generate and establish the ‘structure-cytotoxicity’ relationship models against 278 cancer cell lines and 27 normal cell lines of humans [31]. The chemical structure of each compound in the form of SMILES was submitted to CLC-Pred to predict the cytotoxic activity.

### 5.14. Statistical Analysis

A statistical analysis was completed using Prism v5.0 (GraphPad Software Inc., San Diego, California, USA). Data are stated as mean ± SEM (standard error of mean). A student’s t-test or ANOVA followed by Post Hoc Tukey’s test were used for analyzing the cytotoxicity data. To determine the correlation between variables, a linear regression was carried out. Statistical significance was considered when *p* < 0.05.

## Figures and Tables

**Table 1:** The in silico physicochemical, bioactivity, pharmacokinetic and toxicity profiles of the compounds isolated from *Fusarium oxysporum*.

Parameter	Compound 1	Compound 2	Compound 3	Compound 4	Compound 5
Physicochemical properties					
Molecular Weight, MW	428.65	444.65	122.12	304.38	783.95
Fraction Csp3	0.82	0.82	0.00	0.50	0.47
Num. rotational bonds	4	4	1	6	9
Molar refractivity, MR	129.35	130.55	33.85	87.57	228.14
TPSA	57.53	77.76	37.30	66.76	139.83
Num. H-bond acceptors	3	4	2	4	9
Num. H-bond acceptors	2	3	1	2	0
Lipophilicity, LogP_O/W_: iLogP XLogP3 MLogP WLogP SILICOS-IT Consensus value	4.36 5.93 4.52 5.70 5.66 5.24	4.02 4.48 3.67 4.82 5.17 4.43	0.99 1.35 0.79 1.20 1.52 1.17	3.45 5.29 2.97 3.88 4.40 4.00	5.30 8.42 3.14 3.77 5.43 5.21
Water solubility:					
Log S (ESOL)	Moderately soluble	Moderately soluble	Very soluble	Moderately soluble	Poorly soluble
Log S (SILICOS-IT)	Moderately soluble	Moderately soluble	Soluble	Moderately soluble	Insoluble
Log S (Ali)	Poorly soluble	Moderately soluble	Very soluble	Poorly soluble	Insoluble
Drug-likeness:					
Lipinski’s filter	No violation	No violation	No violation	No violation	2 violations (MW > 500; N or O > 10)
Ghose filter	2 violations (WLogP > 5.6; atom num. > 70)	2 violations (MR > 130; atom num. > 70)	3 violations (MW < 160, MR < 40; atom num. < 20)	No violation	3 violations (MW > 480; MR > 130; atom num. > 70)
Veber’s filter	No violation	No violation	No violation	No violation	No violation
Egan’s filter	No violation	No violation	No violation	No violation	1 violation (TPSA > 131.6)
Muegge’s filter	1 violation (XLogP3 > 5)	No violation	1 violation (MW < 200)	1 violation (XLogP3 > 5)	2 violations (MW > 600; XLogP3 > 5)
Bioactivity					
GPCR ligand	0.11	0.16	−2.38	0.23	−1.70
Nuclear receptor ligand	0.75	0.91	−1.93	0.49	−2.60
Kinase inhibitor	−0.44	−0.41	−2.37	−0.25	−2.58
Protease inhibitor	0.02	0.12	−2.80	−0.02	−1.07
Enzyme inhibitor	0.51	0.59	−1.75	0.45	−2.23
Ion channel modulator	0.07	0.17	−1.51	0.07	−2.98
Pharmacokinetic properties					
GI absorption	High	High	High	High	Low
BBB permeant	No	No	Yes	Yes	No
Skin permeation, log Kp (cm/s)	−4.7	−5.83	−6.09	−4.4	−5.1
P-gp substrate	No	Yes	No	No	Yes
Bioavailability	0.55	0.55	0.55	0.55	0.17
CYP1A2 inhibitor	No	No	No	Yes	No
CYP2C19 inhibitor	No	No	No	No	No
CYP2C9 inhibitor	No	No	No	Yes	No
CYP2D6 inhibitor	No	No	No	No	No
CYP3A4 inhibitor	No	No	No	No	No
Toxic properties					
Mutagenic effect	Absent	Absent	Highly present	Moderately present	Absent
Tumorigenic effect	Absent	Absent	Absent	Absent	Absent
Irritant effect	Absent	Absent	Highly present	Absent	Absent
Reproductive effect	Absent	Absent	Absent	Absent	Absent
Max. tolerated dose in human, Log (mg/kg/day)	−0.389	−0.414	1.246	−0.318	0.188
Hepatotoxicity	Yes	Yes	No	No	Yes
hERG I inhibitor	No	No	No	No	No
hERG II inhibitor	No	No	No	Yes	Yes

Predicted data based on SwissADME, Molinspiration, Osiris Property Explorer and pkCSM web service. Here, TPSA = topological polar surface area; N or O = NH or OH = number of NH and OH; BBB = blood brain barrier; GPCR = G-protein coupled receptor; GI = gastrointestinal.

**Table 2:** The in silico prediction of the cytotoxic activities of the compounds isolated from *F. oxysporum* on cell lines.

Compounds	Cell Line	Pa	Pi
Compound **1**	Colon adenocarcinoma (HCC 2998) Ovarian adenocarcinoma (OVCAR-5) Lung carcinoma (DMS-114)	0.633 0.616 0.610	0.009 0.013 0.009
Compound **2**	Lung carcinoma (DMS-114) Small cell lung carcinoma (NCI-H187) Colon adenocarcinoma (HCC 2998)	0.584 0.501 0.476	0.012 0.014 0.024
	Embryonic lung fibroblast (WI-38 VA13)	0.413	0.033
Compound **3**	Oligodendroglioma (Hs 683) Small cell lung carcinoma (NCI-H187) Non-small cell lung carcinoma (HOP-18)	0.664 0.592 0.431	0.014 0.005 0.022
Compound **4**	Melanoma (A2058) Small cell lung carcinoma (NCI-H187) Lung carcinoma (DMS-114)	0.499 0.488 0.448	0.014 0.017 0.072
Compound **5**	Glioblastoma (SF-268) Breast adenocarcinoma (MDA-MB-231) Pancreatic carcinoma (MIA PaCa-2)	0.661 0.553 0.533	0.008 0.020 0.007

Pa = probability of activity and Pi = probability of inactivity.

## Data Availability

The data presented in this study are available on request from the corresponding author.

## Supplementary Materials

The following supporting information can be downloaded at: https://www.mdpi.com/article/10.3390/toxins14030159/s1. This section contains figures related to the plant and fungal identification and NMR and mass spectra of the isolated compounds. Figure S1: Herbarium sheets of the *Aglaonema hookerianum* plant deposited in the Bangladesh National Herbarium (BNH). Figure S2: The microscopical characteristics of *Fusarium oxysporum*. A,B: Macroconidia and microconidia; C,D: The measurements of macroconidia and microconidia. Figure S3: The ^1^H NMR spectrum (400 MHz, CDCl3) of compound **1**. Figure S4: The ^13^C NMR spectrum (100 MHz, CDCl_3_) of compound **1**. Figure S5: The ^13^C NMR spectrum (100 MHz, CDCl_3_) of compound **1** (expanded). Figure S6: the ^13^C NMR spectrum (100 MHz, CDCl_3_) of compound **1** (expanded). Figure S7: The ^13^C NMR spectrum (100 MHz, CDCl_3_) of compound **1** (expanded). Figure S8: The ^13^C NMR spectrum (100 MHz, CDCl_3_) of compound **1** (expanded). Figure S9: The ^1^H NMR spectrum (400 MHz, CDCl3) of compound **2**. Figure S10: The ^1^H NMR spectrum (400 MHz, CDCl_3_) of compound **2** (expanded). Figure S11: The ^1^H NMR spectrum (400 MHz, CDCl_3_) of compound **2** (expanded). Figure S12: The ^13^C NMR spectrum (100 MHz, CDCl_3_) of compound **2**. Figure S13: The ^13^C NMR spectrum (100 MHz, CDCl_3_) of compound **2** (expanded). Figure S14: The DEPT-135 spectrum of compound **2**. Figure S15: The HRMS of compound **2**. Figure S16: The ^1^H NMR spectrum (400 MHz, CDCl_3_ with 2 drops of MeOD) of compound **3**. Figure S17: The ^1^H NMR spectrum (400 MHz, CDCl_3_ with 2 drops of MeOD) of compound **3**. (expanded). Figure S18: The ^13^C NMR spectrum (100 MHz, CDCl_3_ with 2 drops of MeOD) of compound **3**. Figure S19: The DEPT-135 spectrum of compound **3**. Figure S20: The ^1^H NMR spectrum (400 MHz, CDCl_3_) of compound **4**. Figure S21: The ^1^H NMR spectrum (400 MHz, CDCl3) of compound **4** (expanded). Figure S22: The ^1^H NMR spectrum (400 MHz, CDCl_3_) of compound **4** (expanded). Figure S23: The ^13^C NMR spectrum (100 MHz, CDCl_3_) of compound **4**. Figure S24: The ^13^C NMR spectrum (100 MHz, CDCl_3_) of compound **4** (expanded). Figure S25: The DEPT-135 spectrum of compound **4**. Figure S26: The COSY spectrum of compound **4**. Figure S27: The HSQC spectrum of compound **4**. Figure S28: The HMBC spectrum of compound **4**. Figure S29: The HRMS spectrum of compound **4**. Figure S30: The ^1^H NMR spectrum (400 MHz, CDCl_3_) of compound **5**. Figure S31: The ^1^H NMR spectrum (400 MHz, CDCl3) of compound **5** (expanded). Figure S32: The ^1^H NMR spectrum (400 MHz, CDCl_3_) of compound **5** (expanded). Figure S33: The ^13^C NMR spectrum (100 MHz, CDCl_3_) of compound **5**. Figure S34: The DEPT-135 spectrum of compound **5.** Figure S35: The COSY spectrum of compound **5**. Figure S36: The HSQC spectrum of compound **5**. Figure S37: The HMBC spectrum of compound **5**. Figure S38: The HRMS spectrum of compound **5**. Figure S39: The X-ray crystallography of compound **5.** Figure S40: The effects of the compounds **1**, **3**, **4** and **5** on Vero cells compared to the control DMSO. All the compounds reduced the cell viability at the concentration of 20.0µM except for compound **1**. Here, control = DMSO, **1** = 3*β*,5*α*-dihydroxy-ergosta-7,22-diene-6-one, **3** = *p*-hydroxybenzaldehyde, **4** = 3-(*R*)-7-butyl-6,8-dihydroxy-3-pent-11-enylisochroman-1-one, **5** = Beauvericin. Figure S41: The outcomes of compound **1** while running in SwissADME. Figure S42: The outcomes of compound 1 while running in the Cell Line Cytotoxicity Predictor (CLC-Pred) online tool. Figure S43: The outcomes of compound **2** while running in SwissADME. Figure S44: The outcomes of compound **3** while running in SwissADME. Figure S45: The outcomes of compound **3** while running in the pkCSM online tool. Figure S46: The outcomes of compound **4** while running in the pkCSM online tool. Figure S47: The outcomes of compound 4 while running in the Molinspiration online tool. Figure S48: The outcomes of compound **5** while running in SwissADME. Figure S49: The outcomes of compound **5** while running in the Cell Line Cytotoxicity Predictor (CLC-Pred) online tool. Figure S50: The outcomes of compound **5** while running in the pkCSM online tool. References [45, 46, 47, 48] are citied in the Supplementat Materials.
