# Supplementary material for: Physicochemical, Pharmacokinetic and Cytotoxicity of the Compounds Isolated from an Endophyte Fusarium oxysporum: In Vitro and In Silico Approaches
Source: Toxins (Basel). 2022 Feb 23;14(3):159. doi: 10.3390/toxins14030159 (PMC8952373; doi:10.3390/toxins14030159)
Supplement: Supplementary file 1 [file toxins-14-00159-s001.zip › toxins-1585218-supplementary.pdf]

toxins

MDPI

Supplementary Materials

Physicochemical, Pharmacokinetic and Cytotoxicity of the Compounds Isolated from an Endophyte *Fusarium oxysporum*: In Vitro and In Silico Approaches

1. The Plant *Aglaonema hookerianum* Schott.

In this study, a fungal endophyte *Fusarium oxysporum*, isolated from petiole of *Aglaonema hookerianum* Schott (Family: Araceae) growing in the forest of Sylhet and Chittagong Hill tracks of Bangladesh has been investigated. It is a herb, stem erect, 40-50 cm or more tall, 1.5-2.0 cm thick, internodes 1.5-3.0 cm long. Leaves petiolate, petiole up to 24 cm long and grows in the shady areas of deep forest. A large number of tribal populations like Chakma, Marma, Murong, Tongchongya, Tripura, Chak, Khasia, Rheyang, Rakhain, Khumi etc live as forest inhabitants in the remote areas throughout Sylhet and the Chittagong Hill Tracts, where there is no or poor modern medical systems of health care. The majority of them are dependent on the traditional system of treatment, which includes various indigenous medicinal plants of those areas [1]. *A. hookerianum,* locally known as Habinishak, is used by the mainstream traditional practitioners of Sylhet district for the treatment of hemorrhoids and arthritis. One tablespoon decoction prepared from the roots of *A. hookerianum* is taken orally twice daily for the treatment of gout. The sap from the root is taken for conjunctivitis and constipation by Chakma community. The leaf extract, applied to the whole body, is used for the treatment of hysteria by Tanchangya community [2]. The petiole of *A. hookerianum* is used in the preparation of 'Shuktani', which is an ethno-medico recipe used in the treatment of stomach disorders like diarrhea, indigestion and dysentery by the Sylheti Bengali Community of Barak Valley, Southern Assam, India [3]. Previous phytochemical study revealed the presence of alkaloid, glycoside, tannin, reducing sugar, saponin and gum in the ethanolic extract of *A. hookerianum*. The extract of this plant also showed potent antibacterial and cytotoxic activities [4].

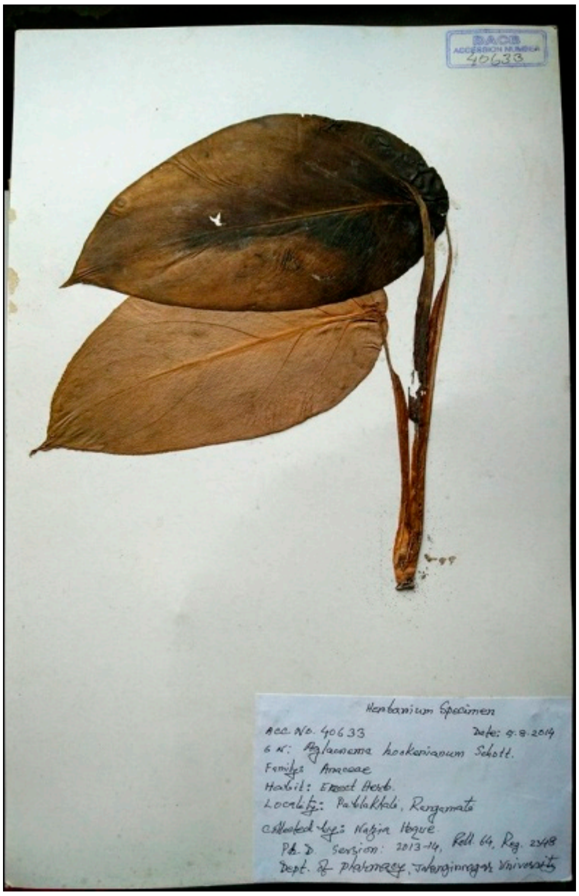

Figure S1. Herbarium sheets of *Aglaonema hookerianum* plant deposited in Bangladesh National Herbarium (BNH).

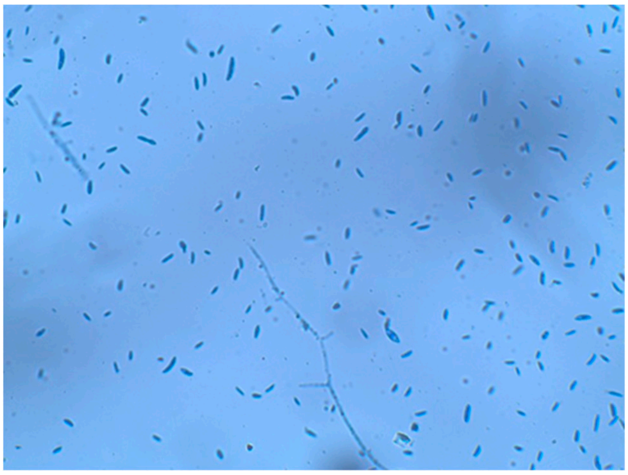

A

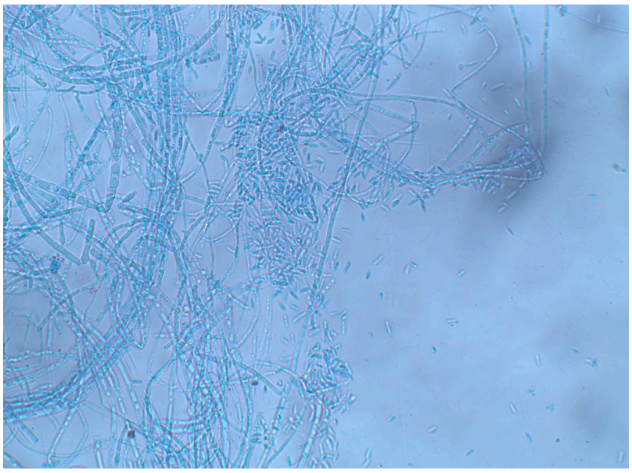

B

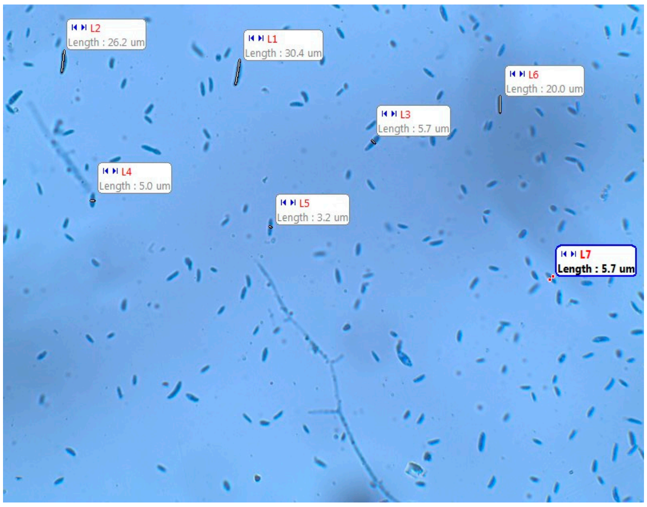

C

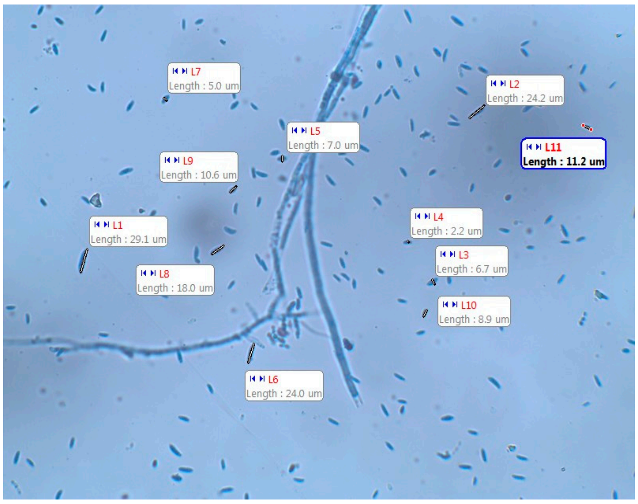

D

Figure S2. Microscopical characters of *Fusarium oxysporum* **A-B**: Macroconidia and microconidia; **C-D**: Measurements of macroconidia and microconidia.

2. NMR and Mass Spectra of Compounds 1–5

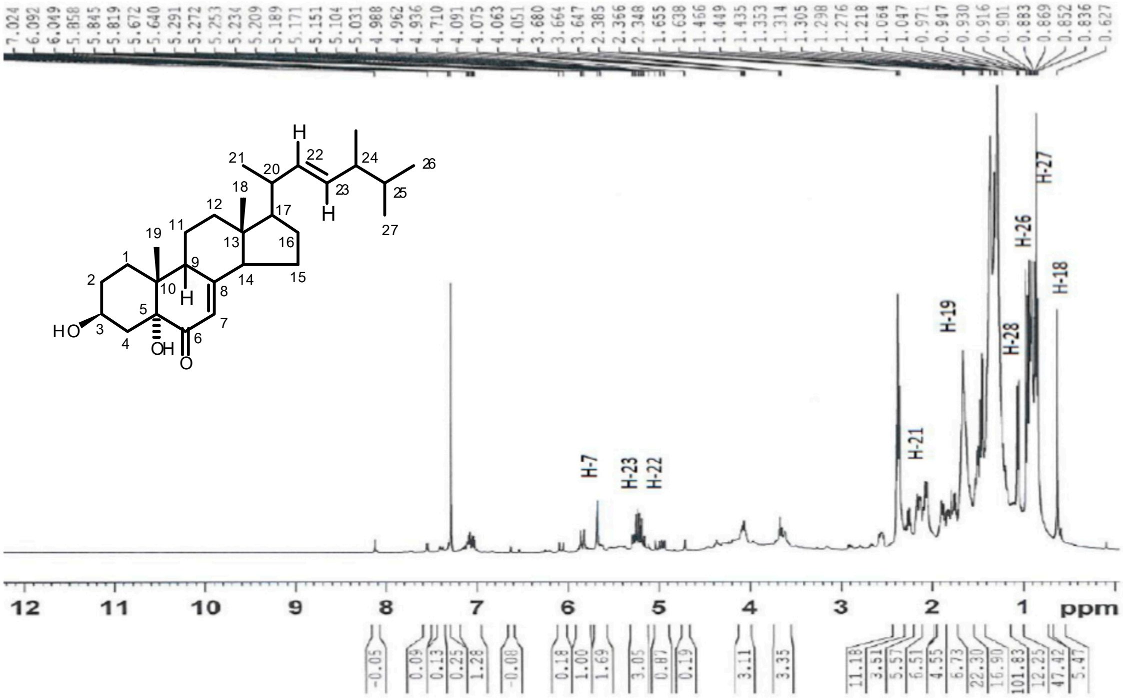

Figure S3. ^{1}H nuclear magnetic resonance (NMR) spectrum (400 MHz, $CDCl_3$) of compound **1.**

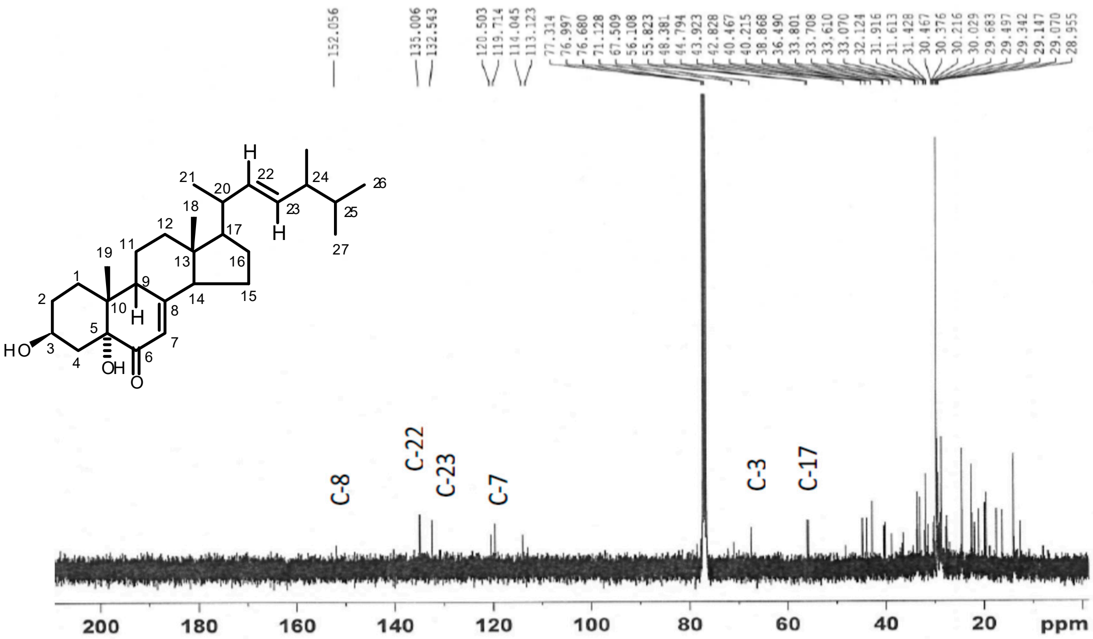

Figure S4. ^{13}C NMR spectrum (100 MHz, $CDCl_3$) of compound 1.

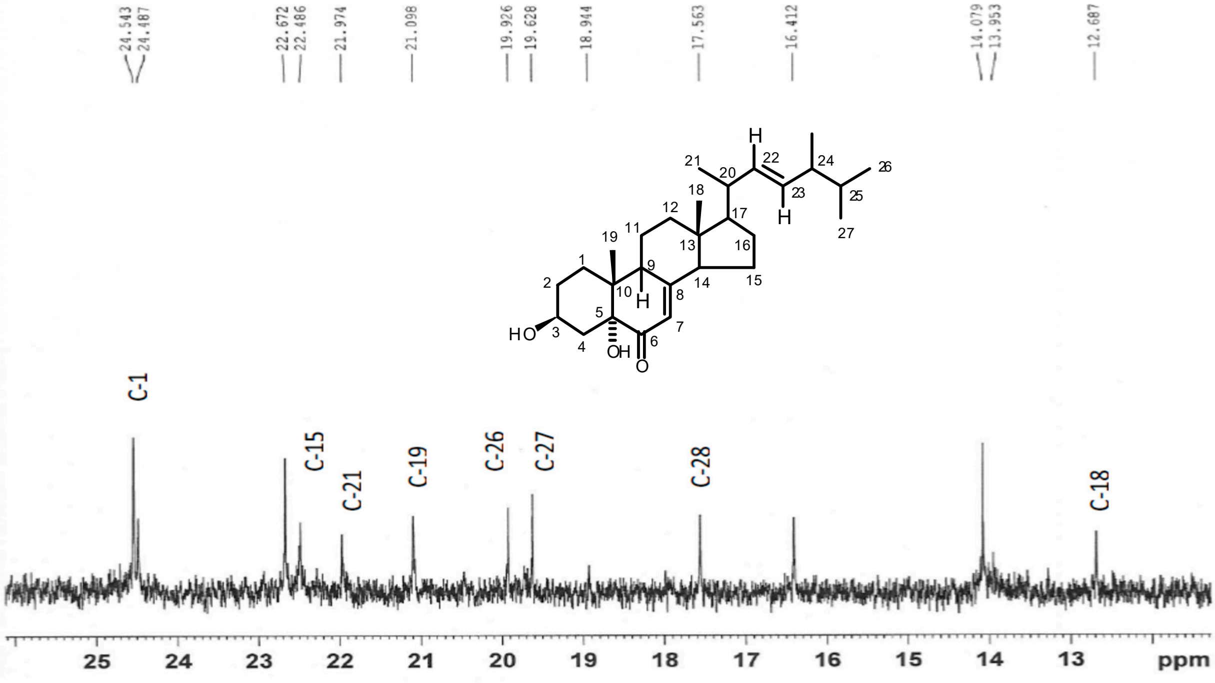

Figure S5. ^{13}C NMR spectrum (100 MHz, $CDCl_3$) of compound **1** (expanded).

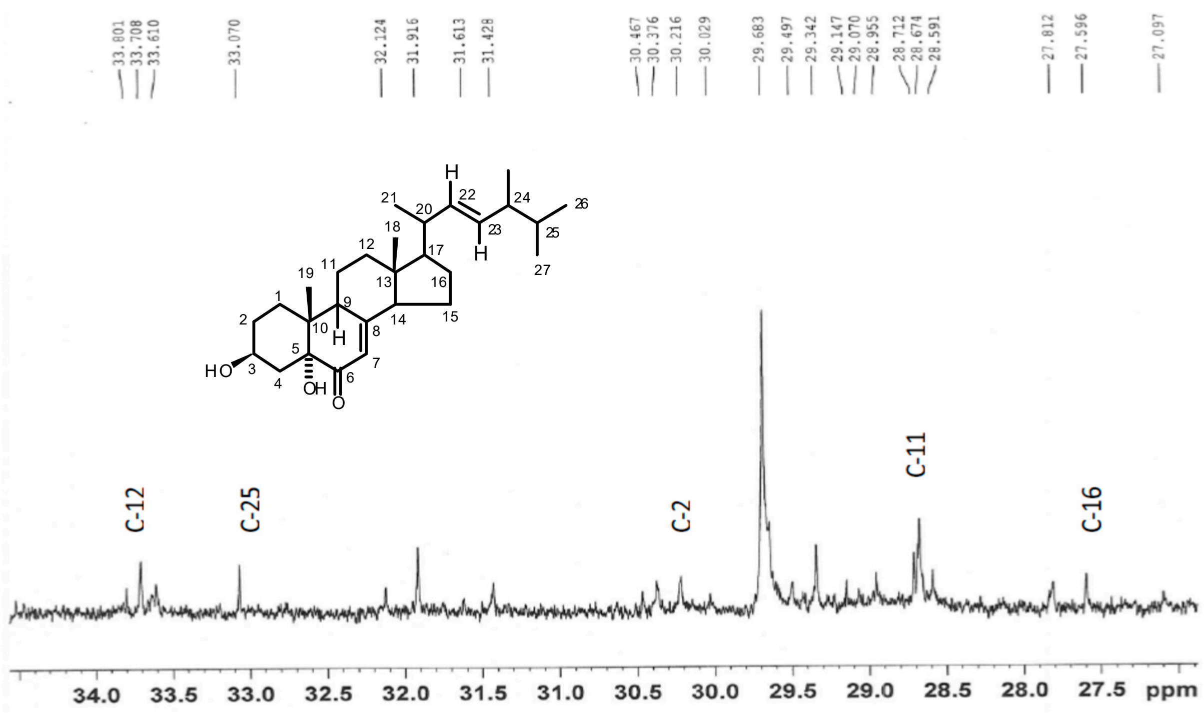

Figure S6. ^{13}C NMR spectrum (100 MHz, $CDCl_3$) of compound **1** (expanded).

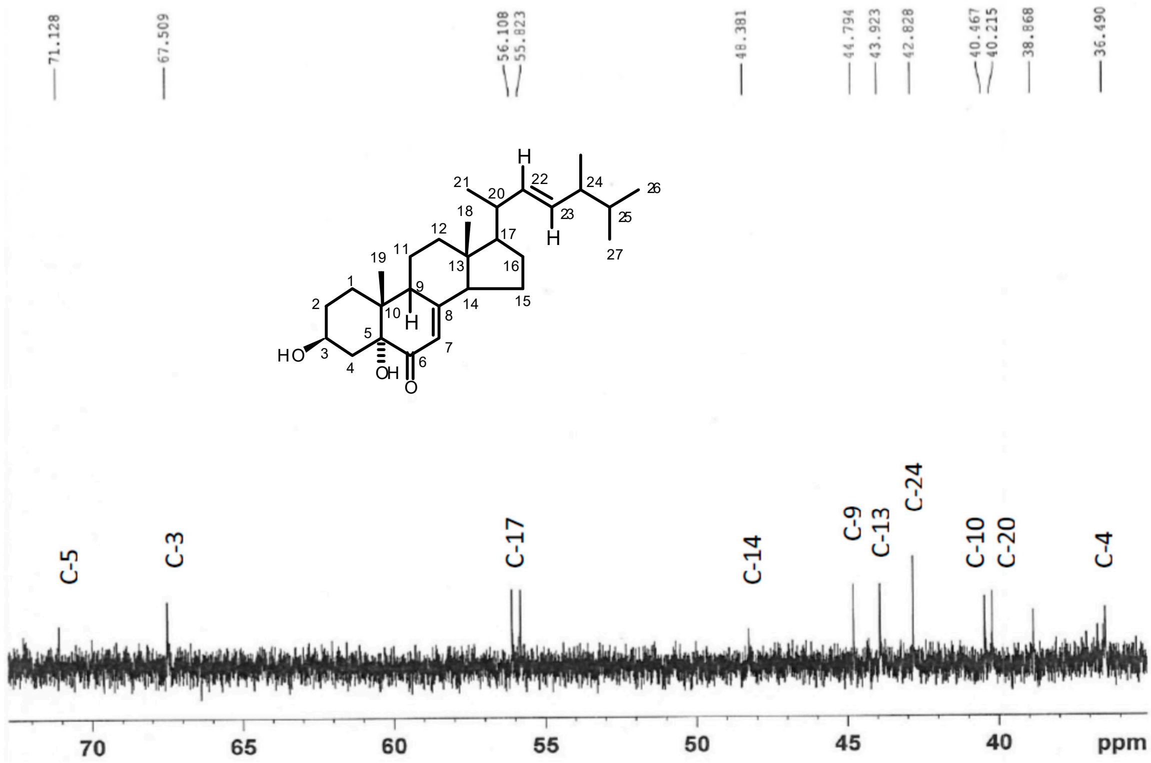

Figure S7. ^{13}C NMR spectrum (100 MHz, $CDCl_3$) of compound **1** (expanded).

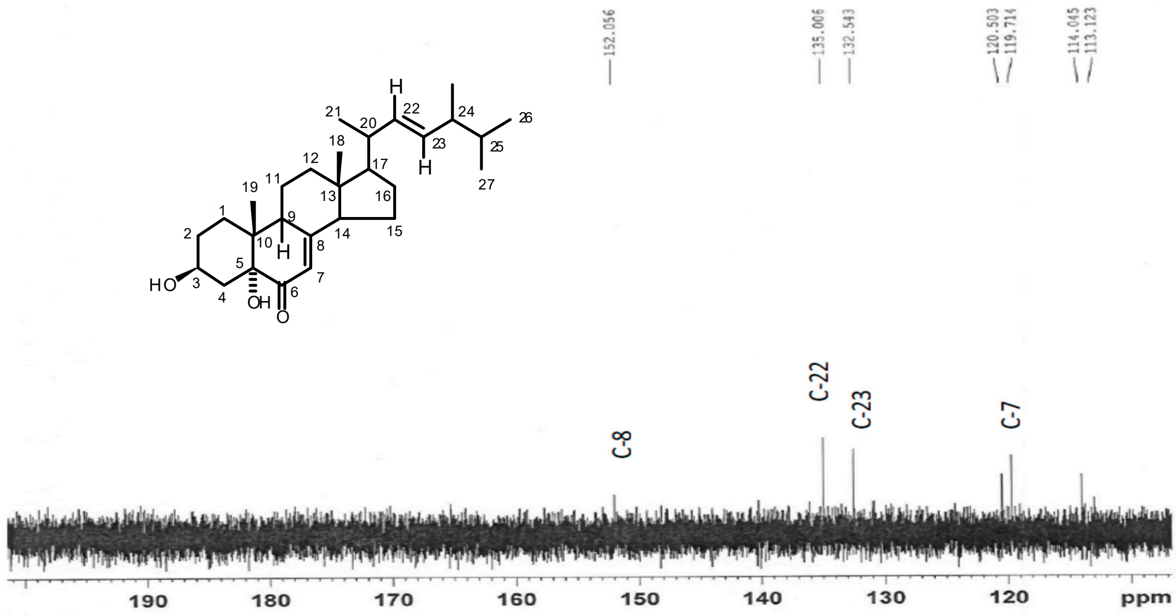

Figure S8. ^{13}C NMR spectrum (100 MHz, $CDCl_3$) of compound **1** (expanded).

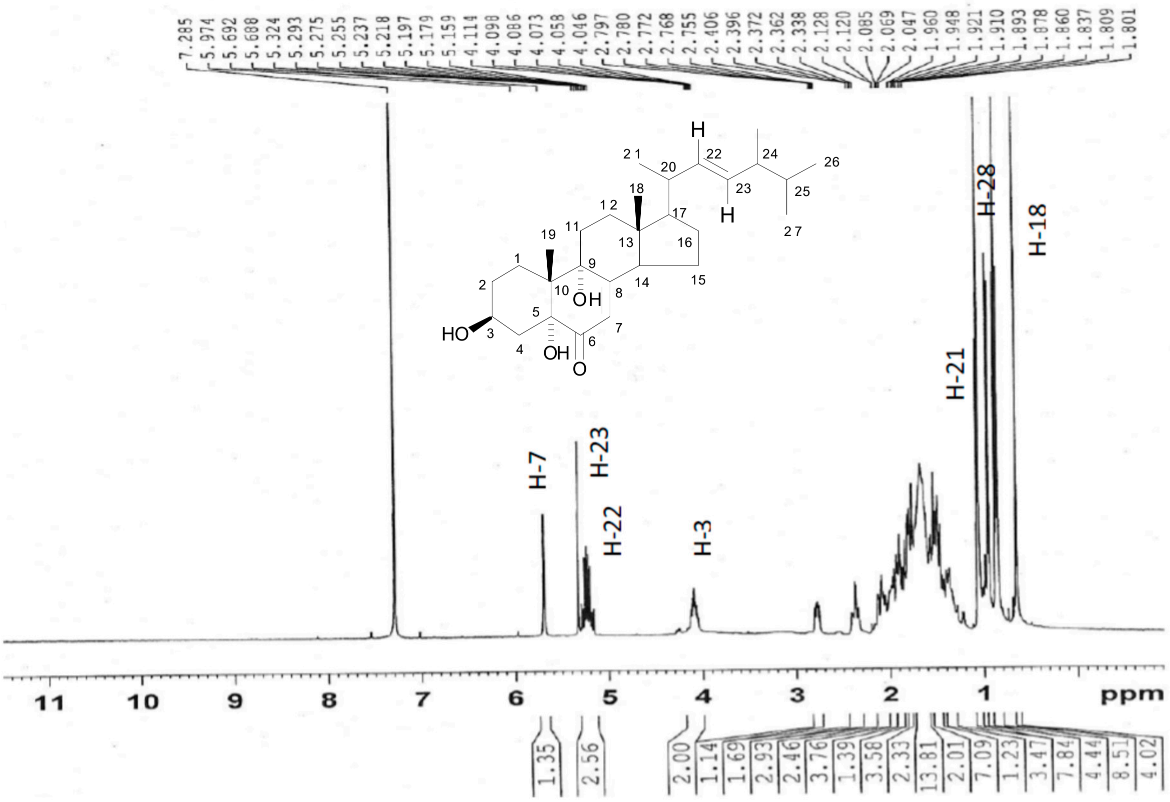

Figure S9. ^{1}H NMR spectrum (400 MHz, $CDCl_3$) of compound **2.**

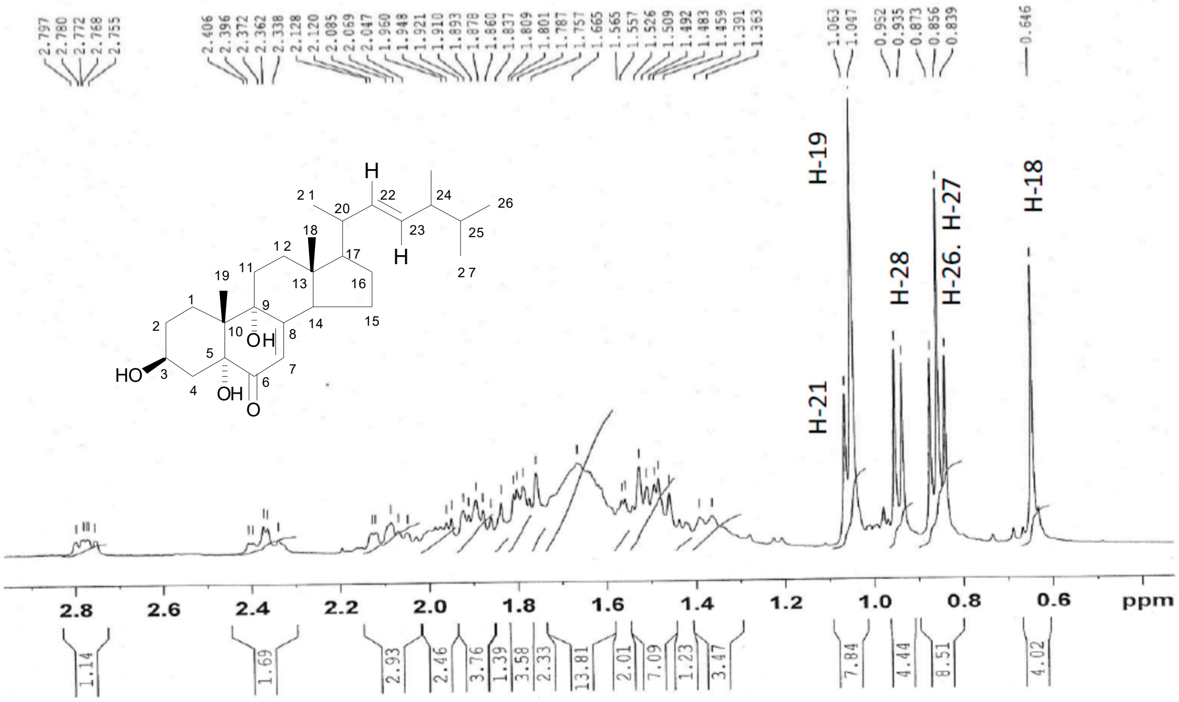

Figure S10. ^{1}H NMR spectrum (400 MHz, $CDCl_3$) of compound 2 (expanded).

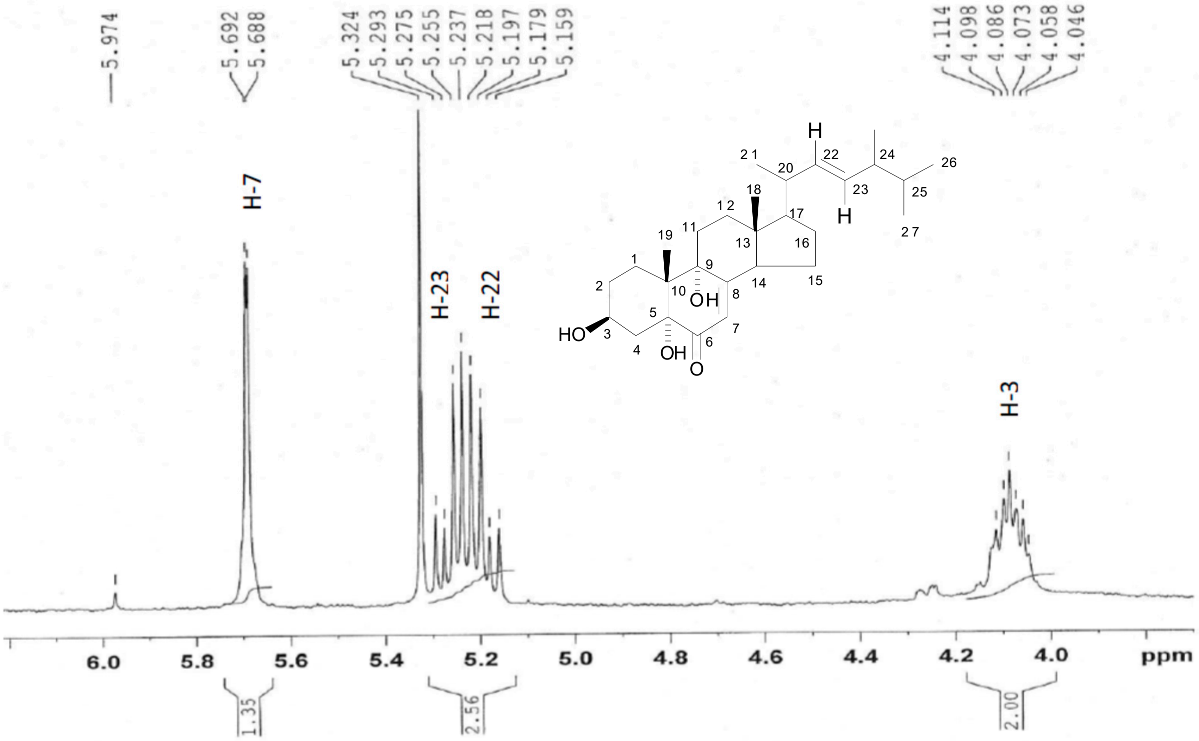

Figure S11. ^{1}H NMR spectrum (400 MHz, $CDCl_3$) of compound **2** (expanded).

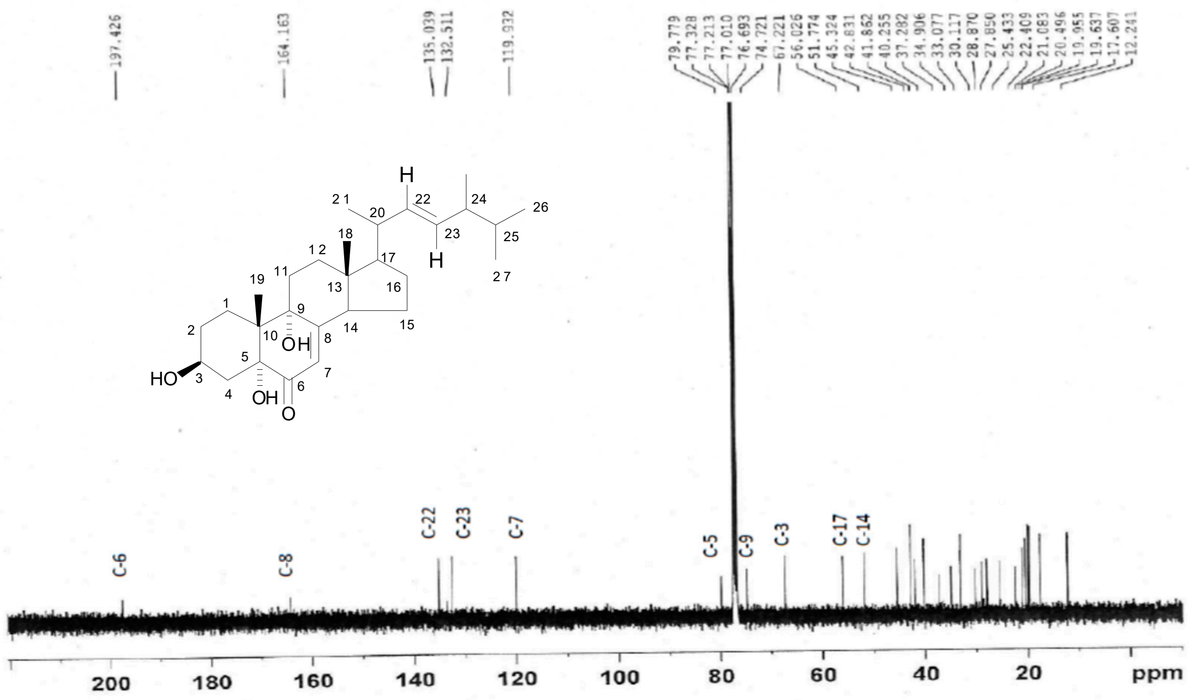

Figure S12. ^{13}C NMR spectrum (100 MHz, $CDCl_3$) of compound **2.**

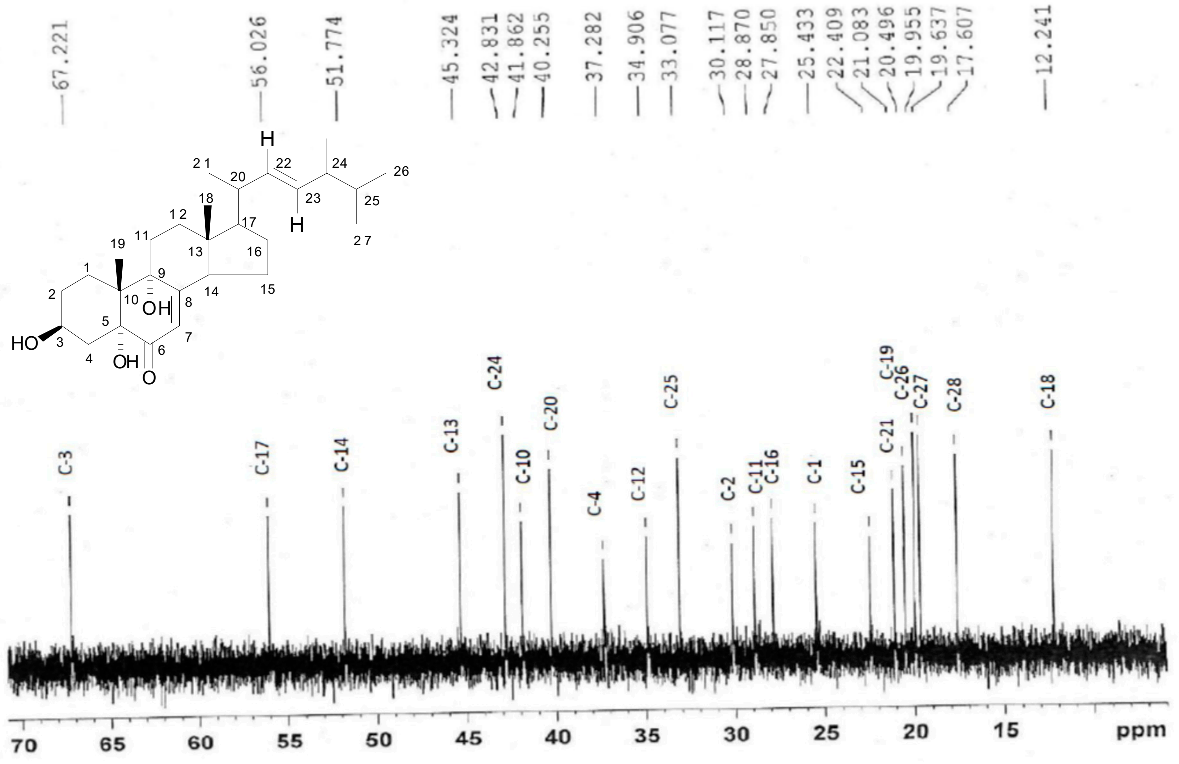

FIGURE

Figure S13. ^{13}C NMR spectrum (100 MHz, $CDCl_3$) of compound **2** (expanded)

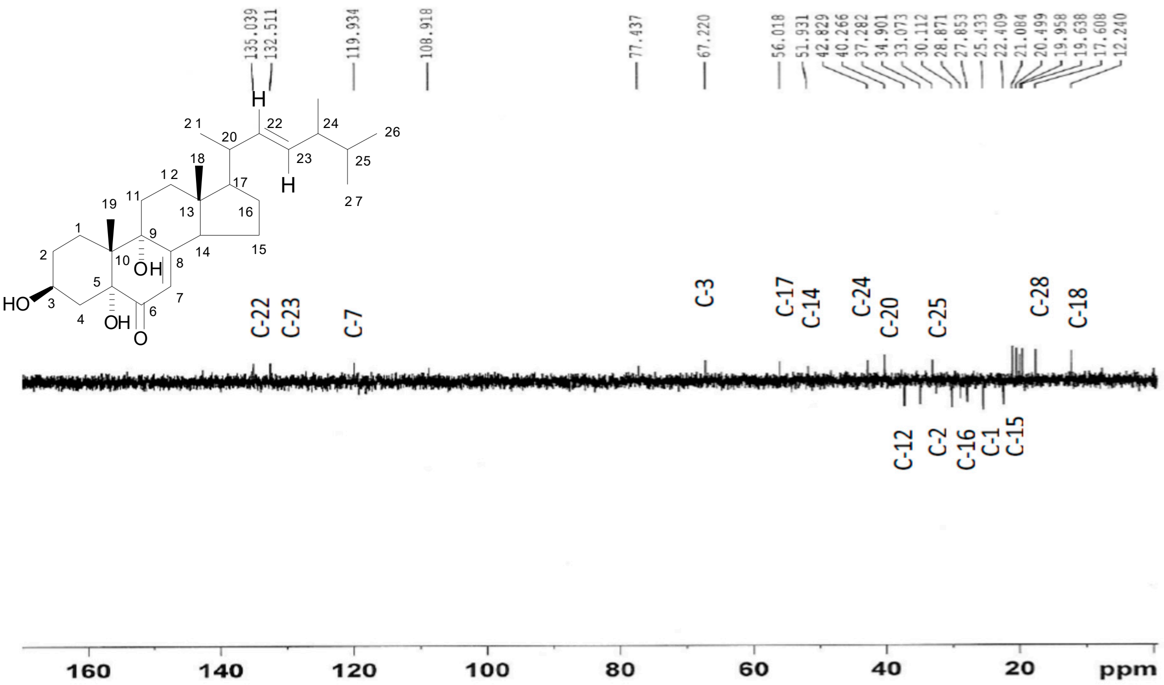

Figure S14. Distortionless enhancement by polarization transfer (DEPT)-135 spectrum of compound **2.**

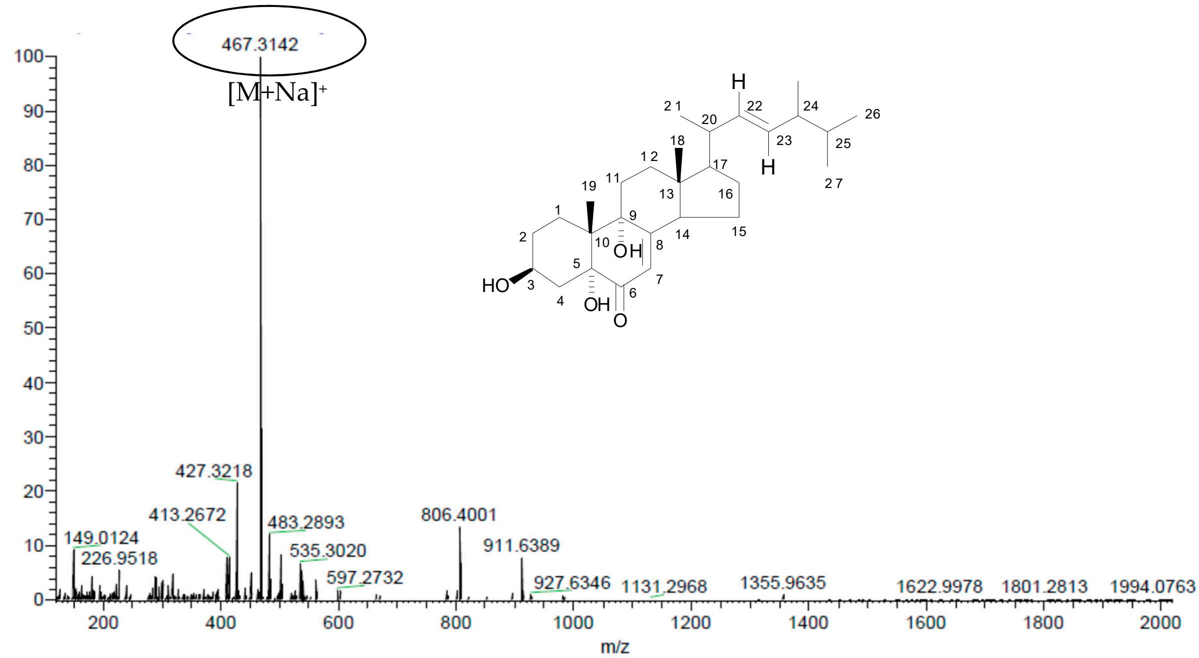

Figure S15. High-resolution mass spectrometry (HRMS) of compound **2.**

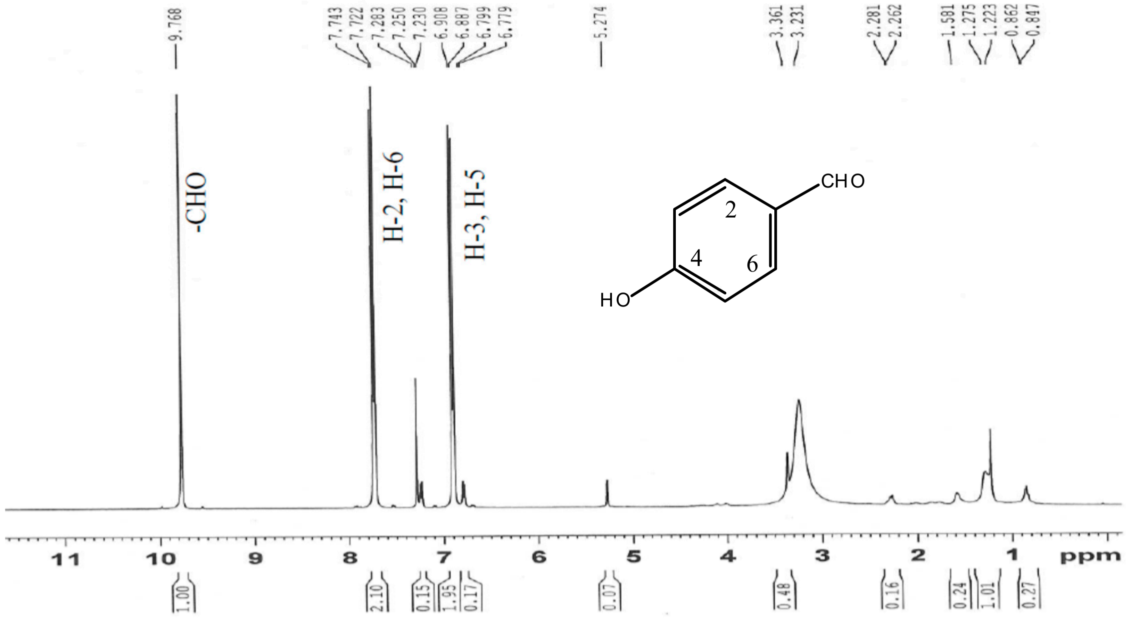

Figure S16. ^{1}H NMR spectrum (400 MHz, $CDCl_3$ with 2 drops of MeOD) of compound **3**.

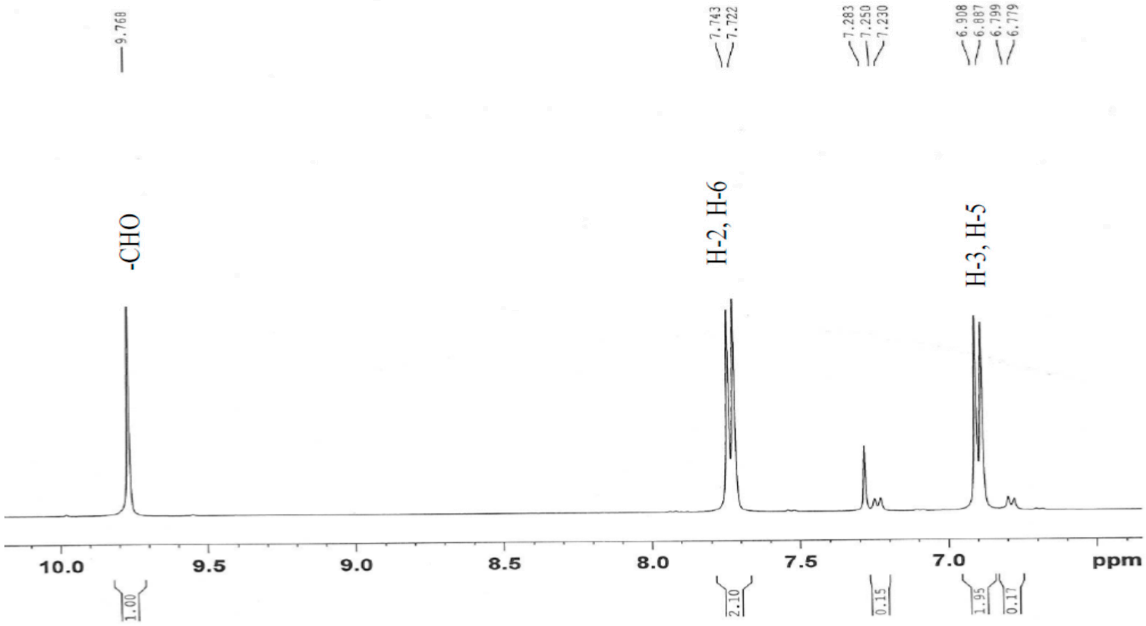

Figure S17. ^{1}H NMR spectrum (400 MHz, $CDCl_3$ with 2 drops of MeOD) of compound **3**. (expanded).

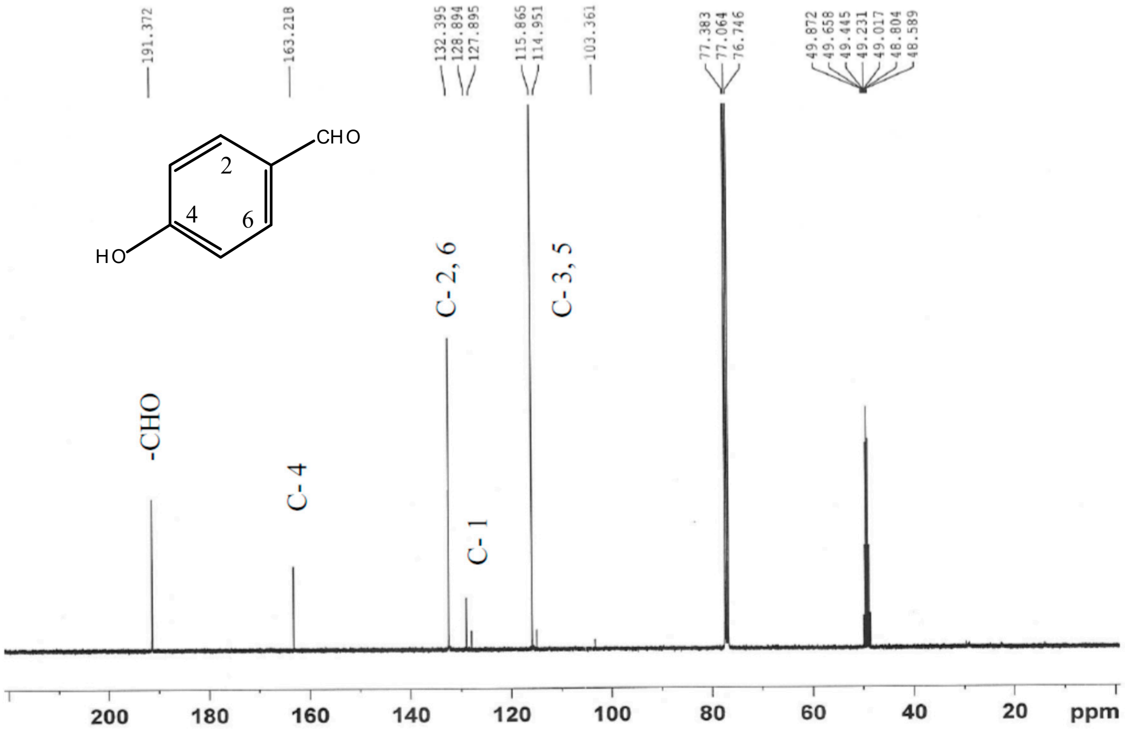

Figure S18. ^{13}C NMR spectrum (100 MHz, $CDCl_3$ with 2 drops of MeOD) of compound **3**.

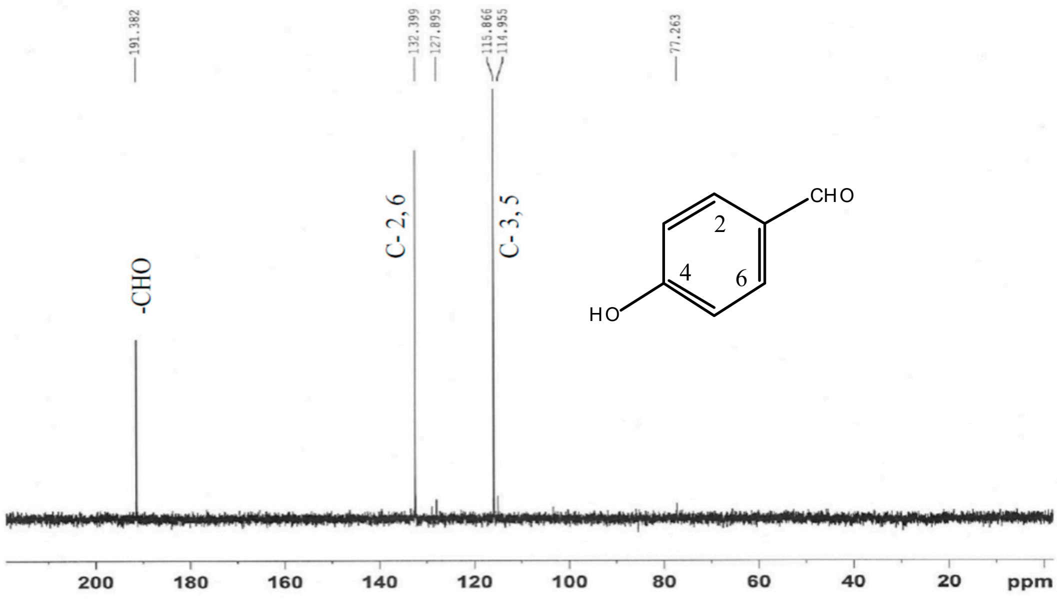

Figure S19. DEPT-135 spectrum of compound **3**.

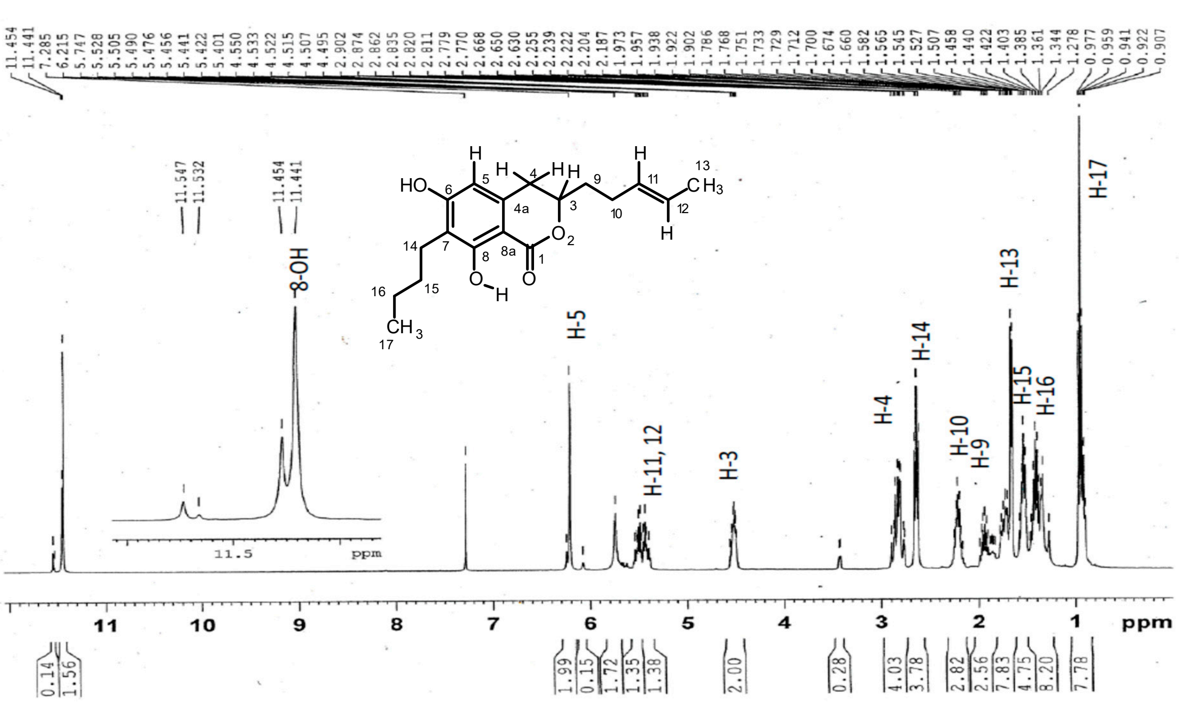

Figure S20. ^{1}H NMR spectrum (400 MHz, $CDCl_3$) of compound **4**.

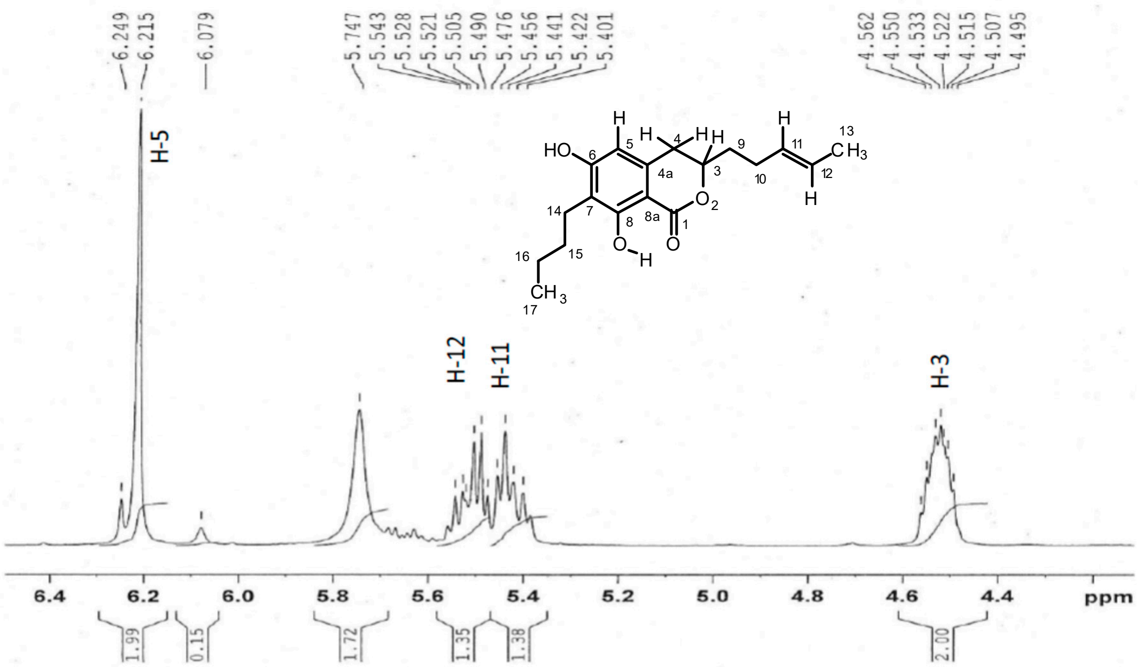

Figure S21. ^{1}H NMR spectrum (400 MHz, $CDCl_3$) of compound **4** (expanded).

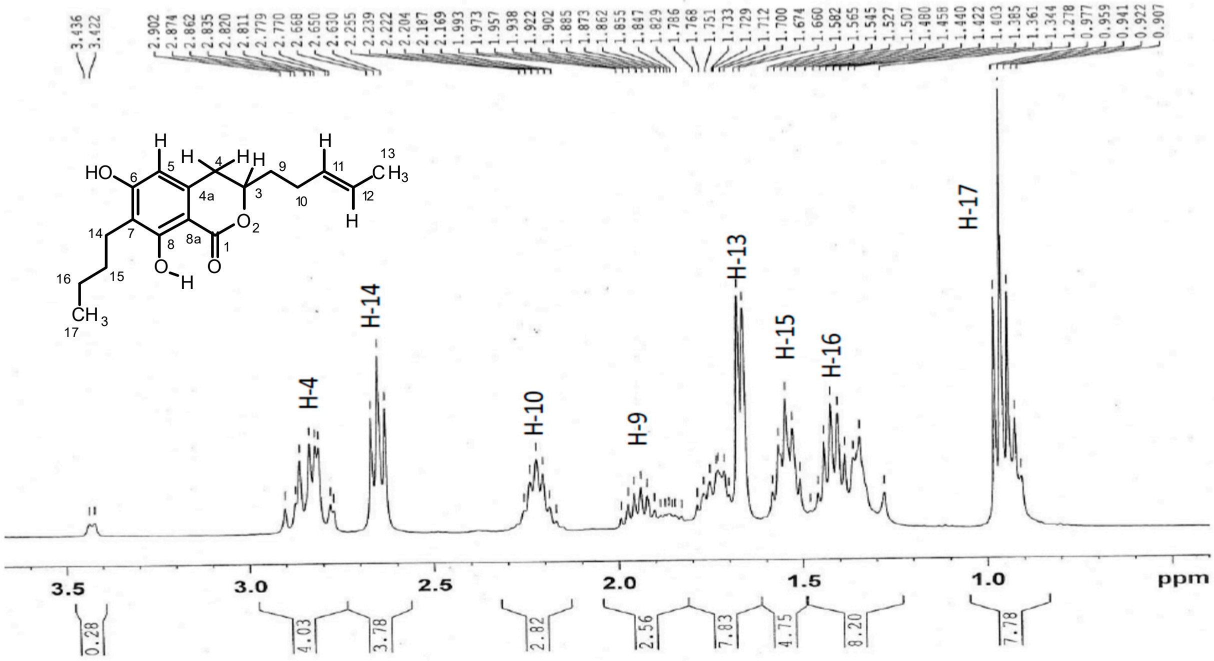

Figure S22. ^{1}H NMR spectrum (400 MHz, $CDCl_3$) of compound **4** (expanded).

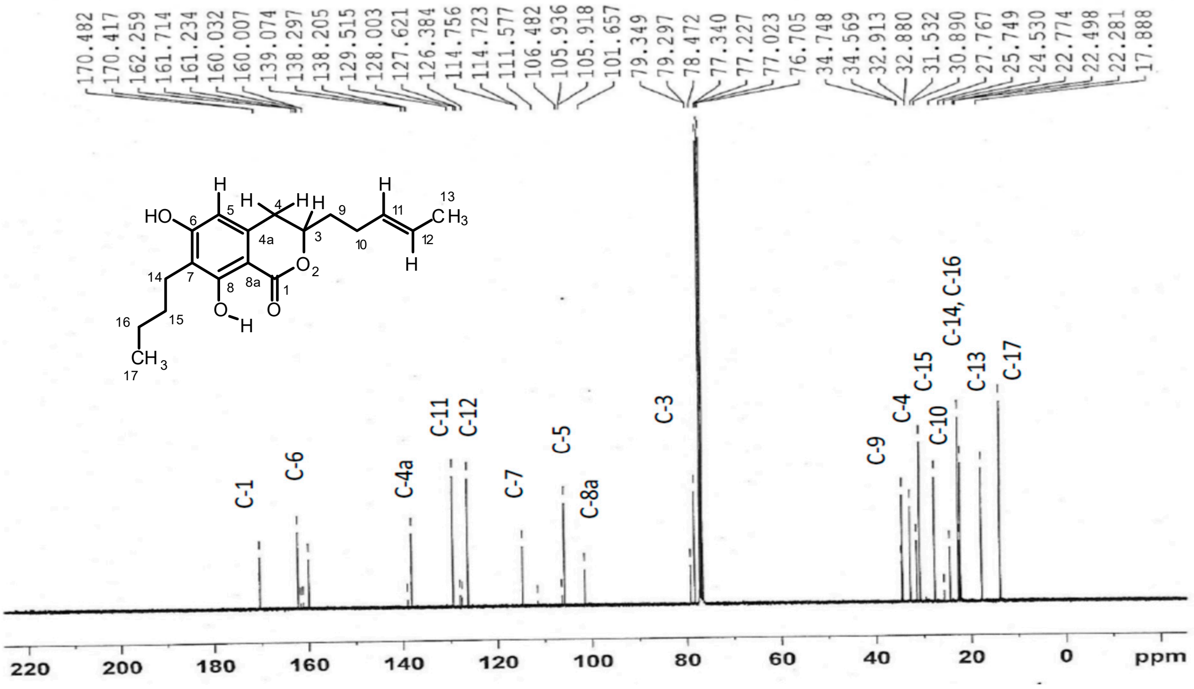

Figure S23. ^{13}C NMR spectrum (100 MHz, $CDCl_3$) of compound **4.**

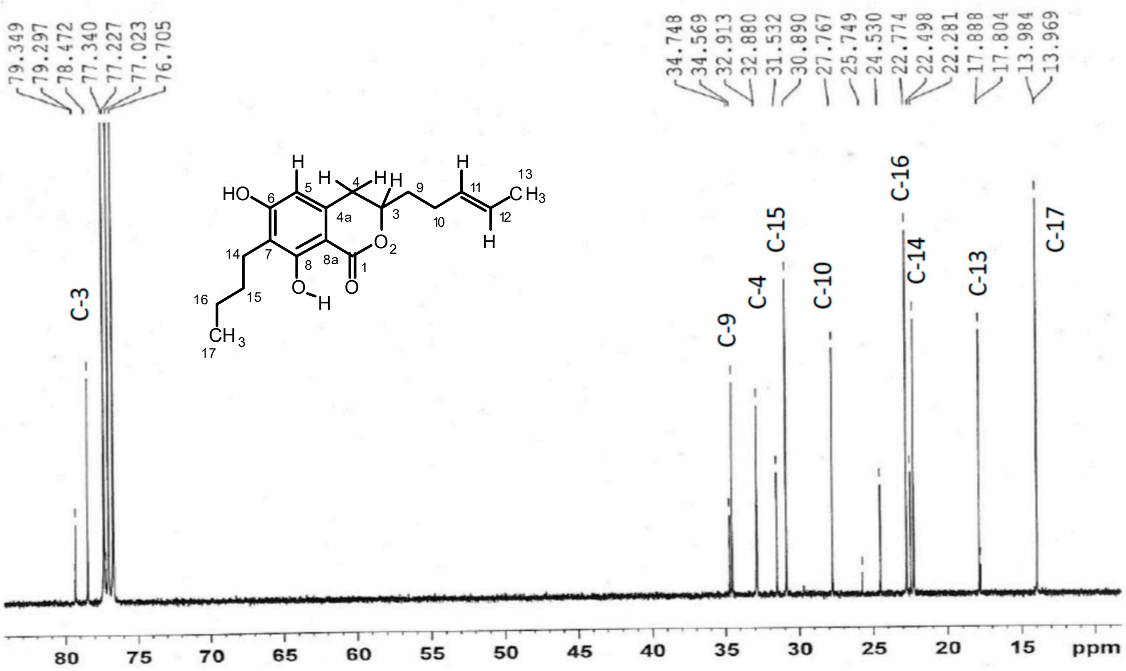

Figure S24. ^{13}C NMR spectrum (100 MHz, $CDCl_3$) of compound **4** (expanded).

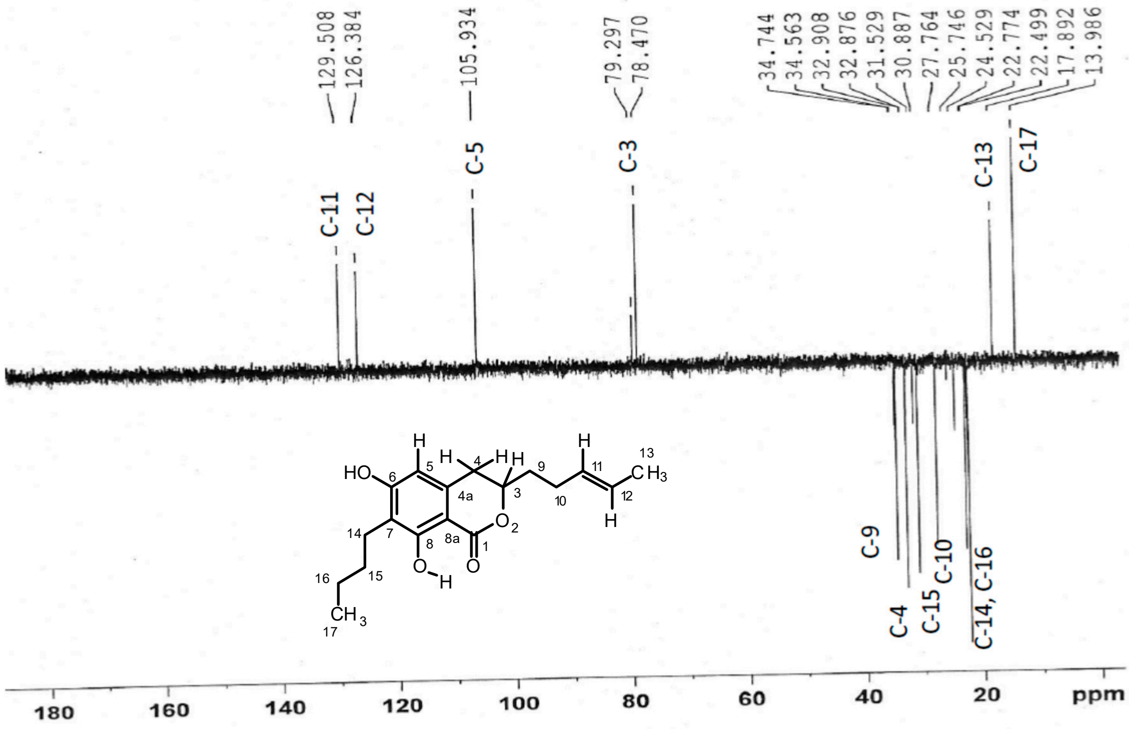

Figure S25. DEPT-135 spectrum of compound **4.**

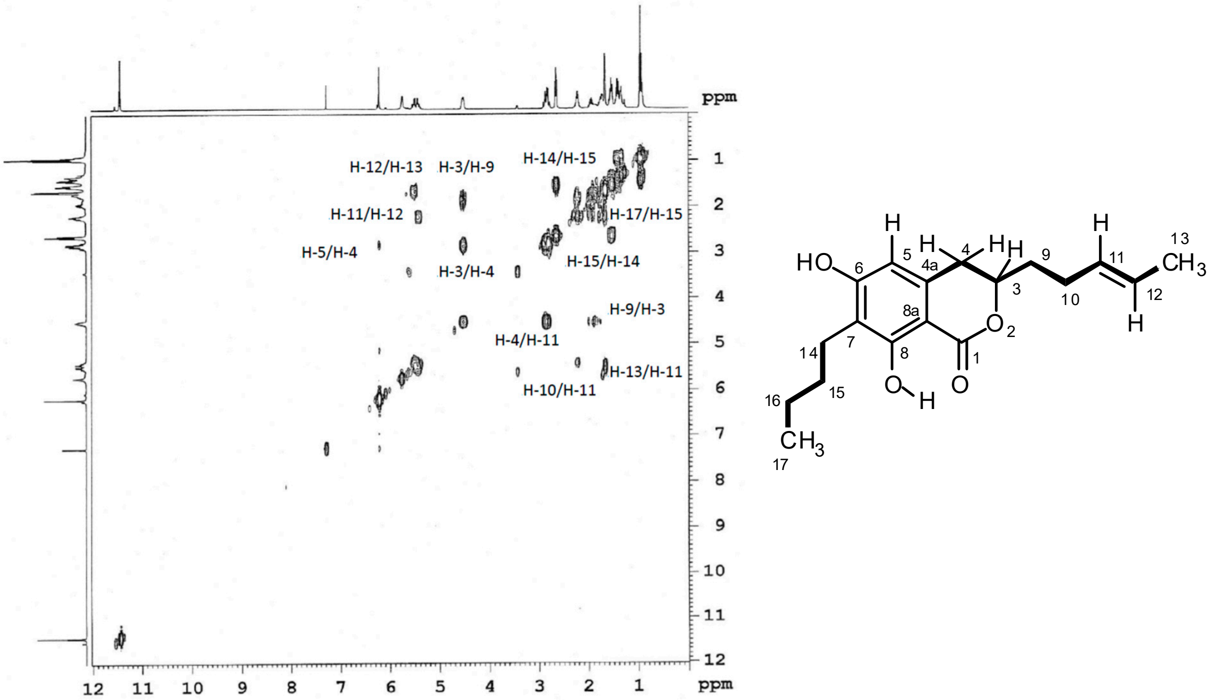

Figure S26. ¹H-¹H Correlation spectroscopy (COSY) spectrum of compound **4.**

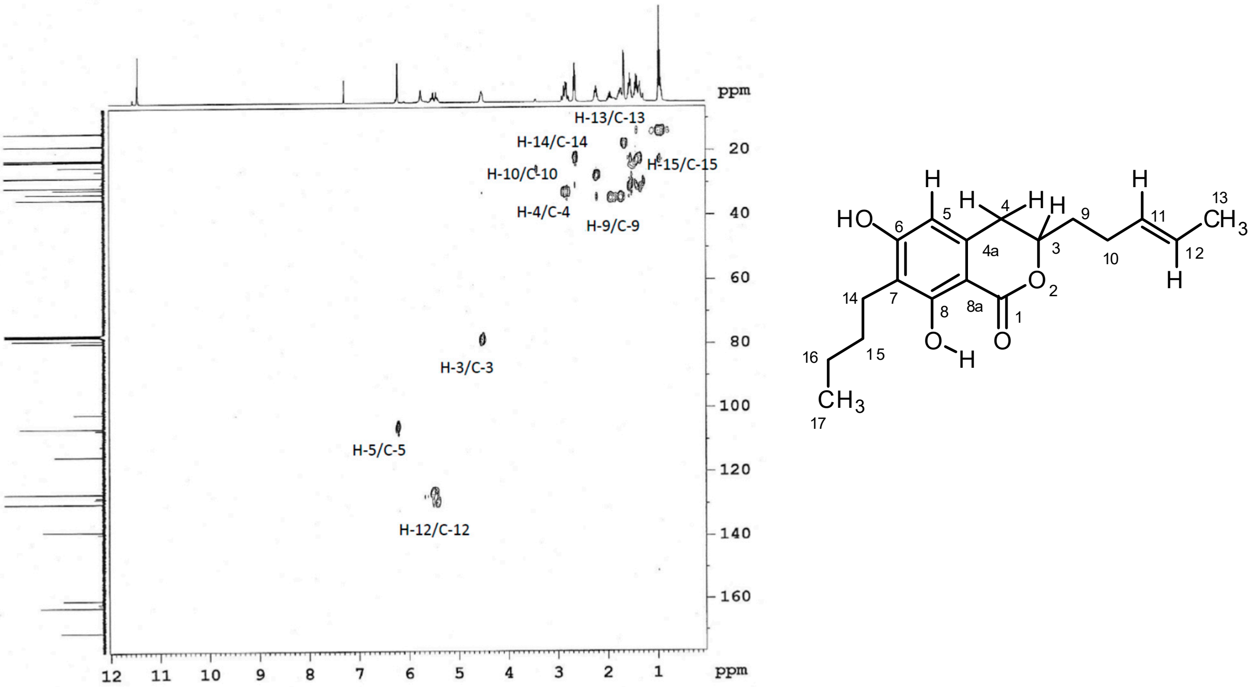

Figure S27. Heteronuclear single quantum coherence or heteronuclear single quantum correlation experiment (HSQC) spectrum of compound **4.**

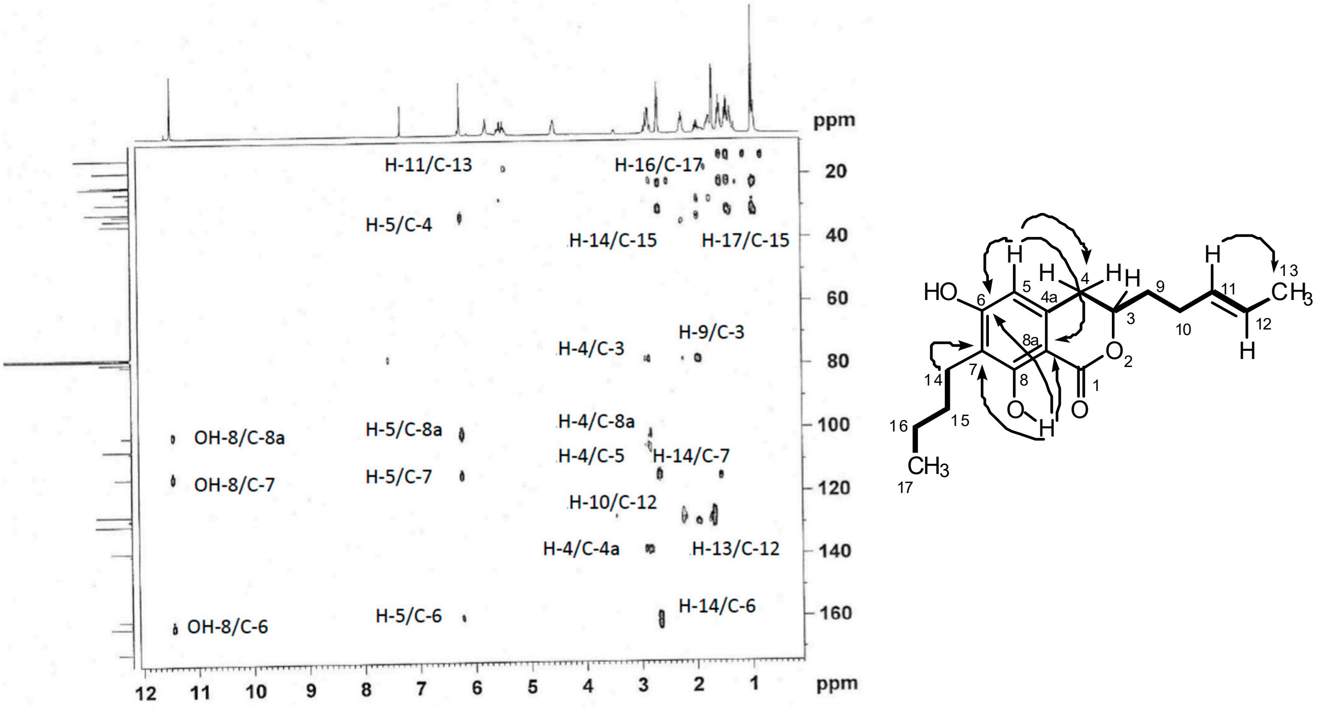

Figure S28. Heteronuclear multiple bond correlation (HMBC) spectrum of compound **4.**

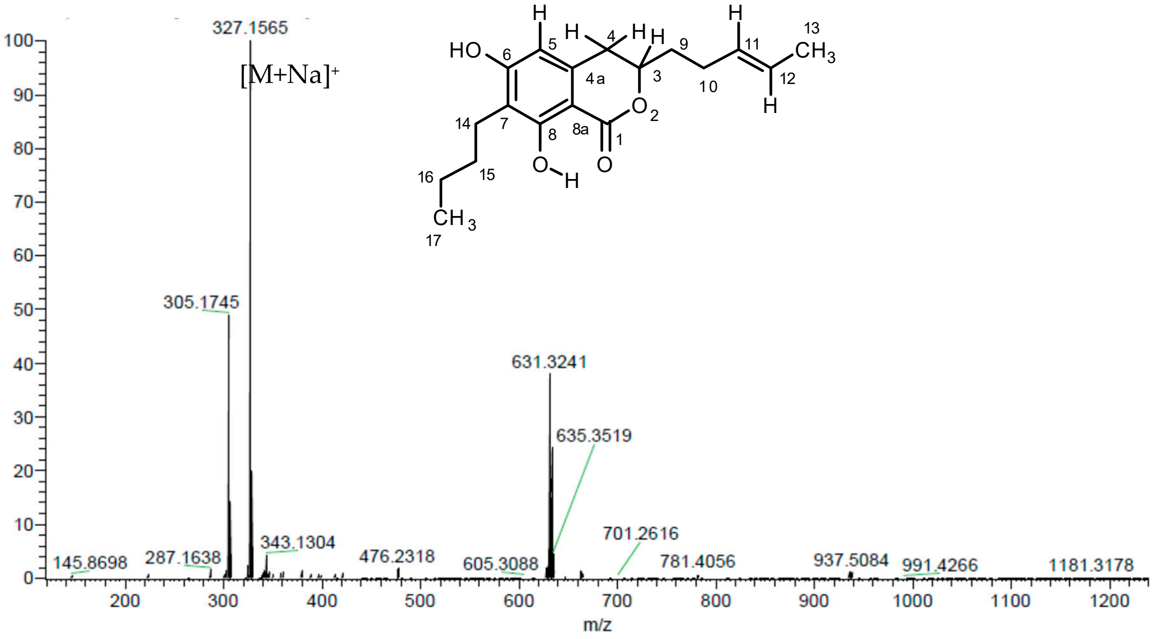

Figure S29. HRMS spectrum of compound **4.**

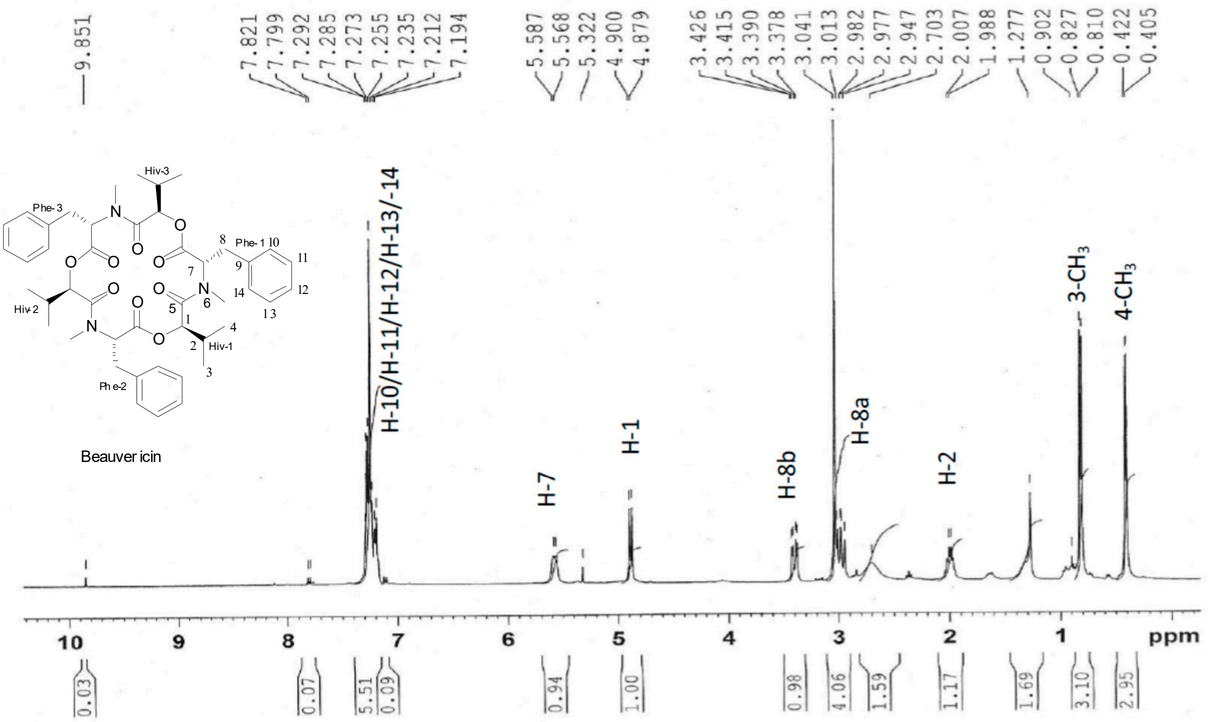

Figure S30. ^{1}H NMR spectrum (400 MHz, $CDCl_3$) of compound **5.**

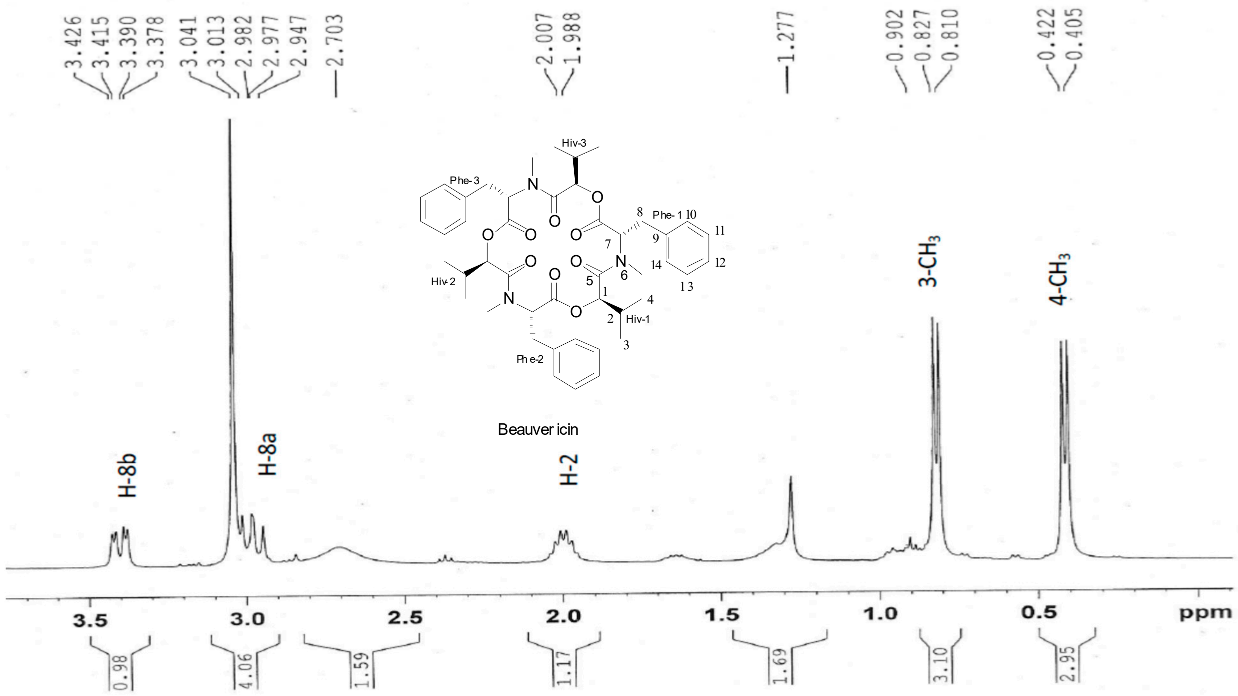

Figure S31. ^{1}H NMR spectrum (400 MHz, $CDCl_3$) of compound **5** (expanded).

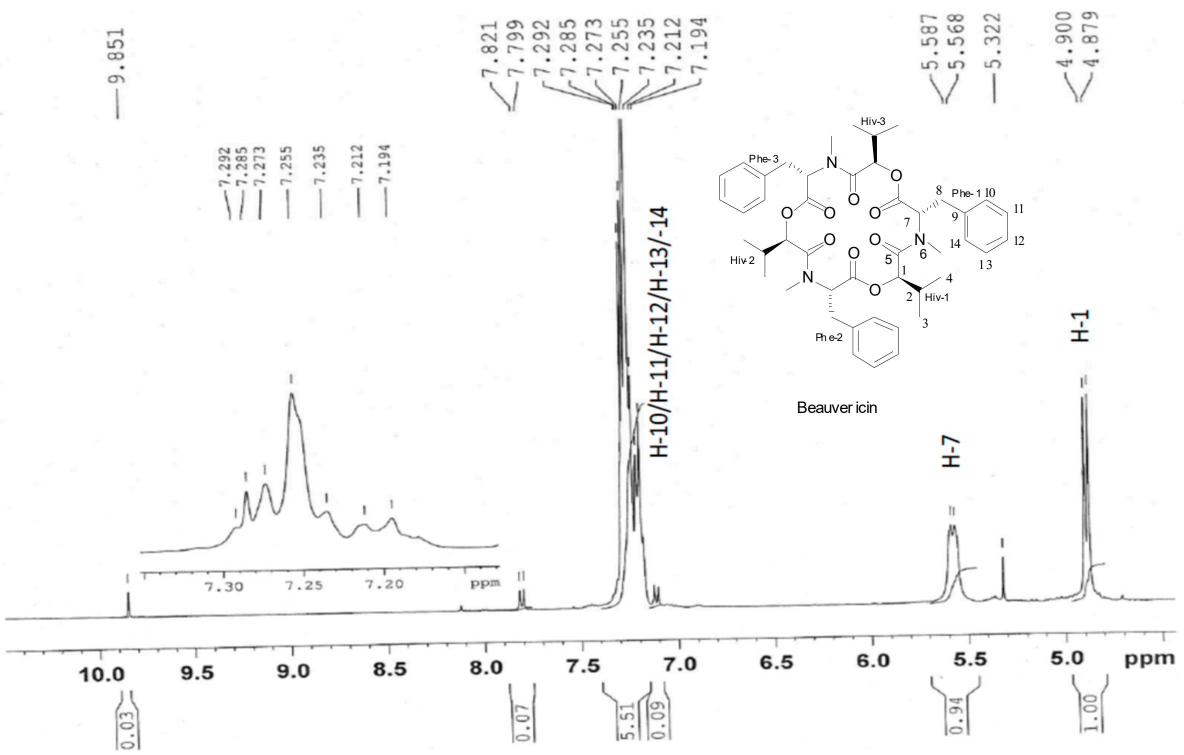

Figure S32. ^{1}H NMR spectrum (400 MHz, $CDCl_3$) of compound **5** (expanded).

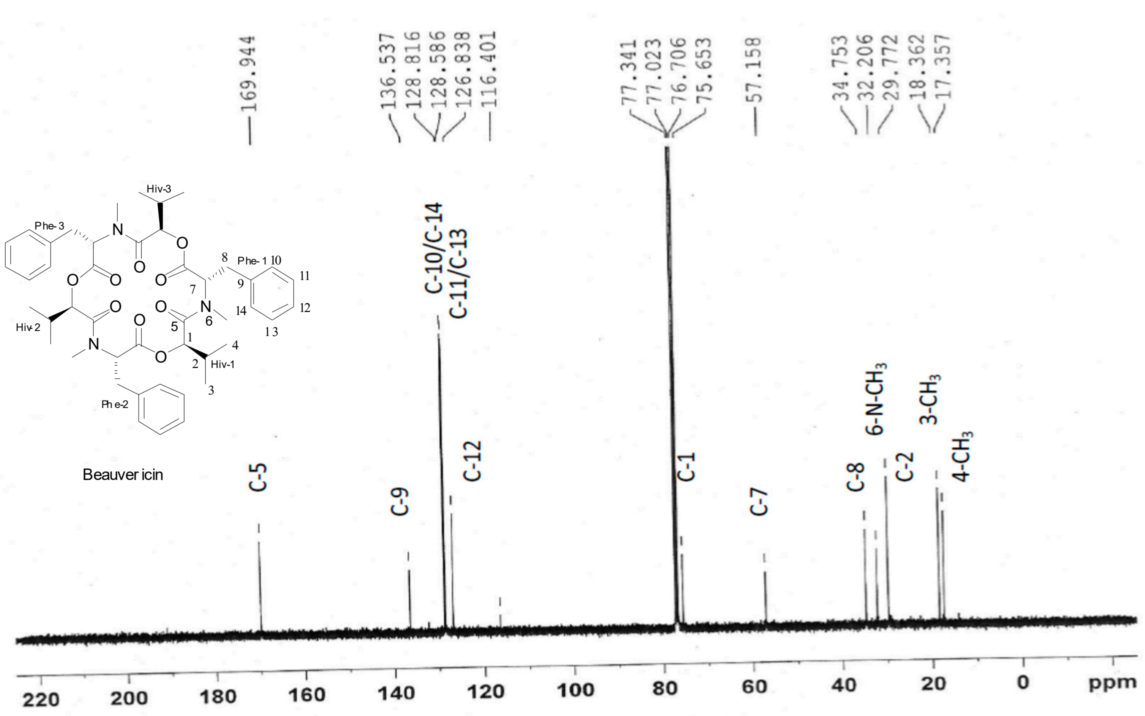

Figure S33. ^{13}C NMR spectrum (100 MHz, $CDCl_3$) of compound **5**.

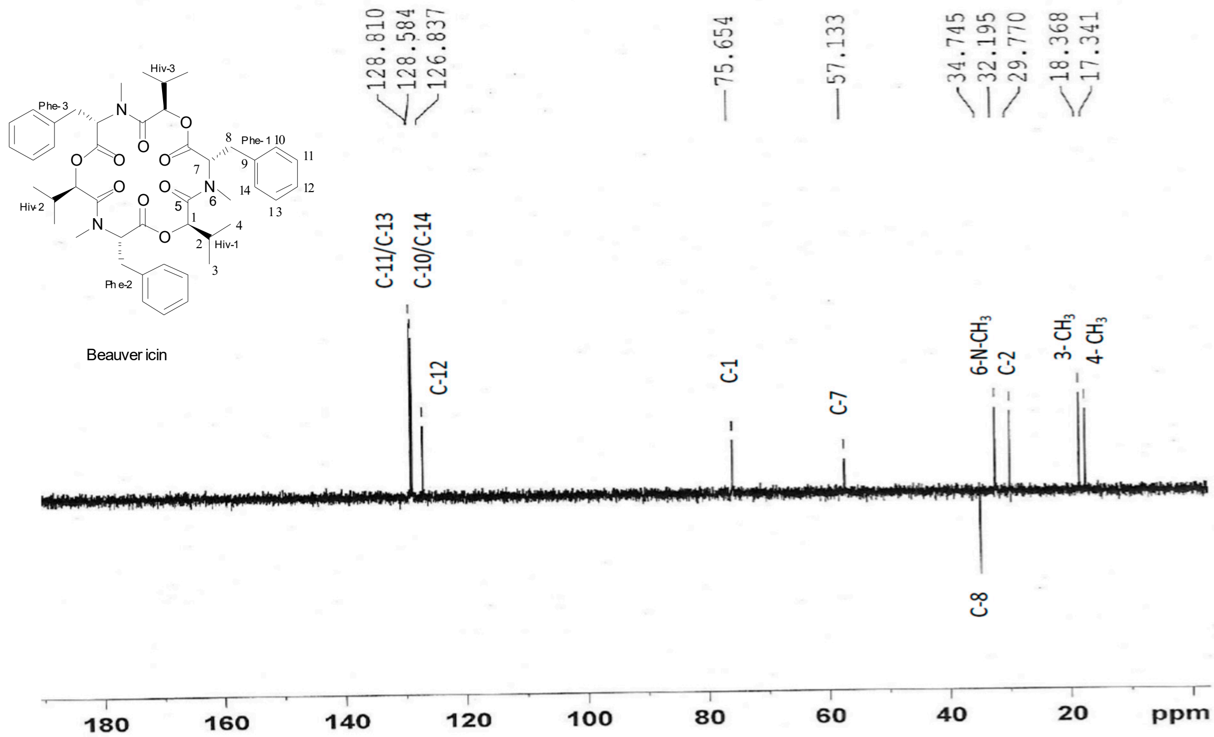

Figure S34. DEPT-135 spectrum of compound **5**.

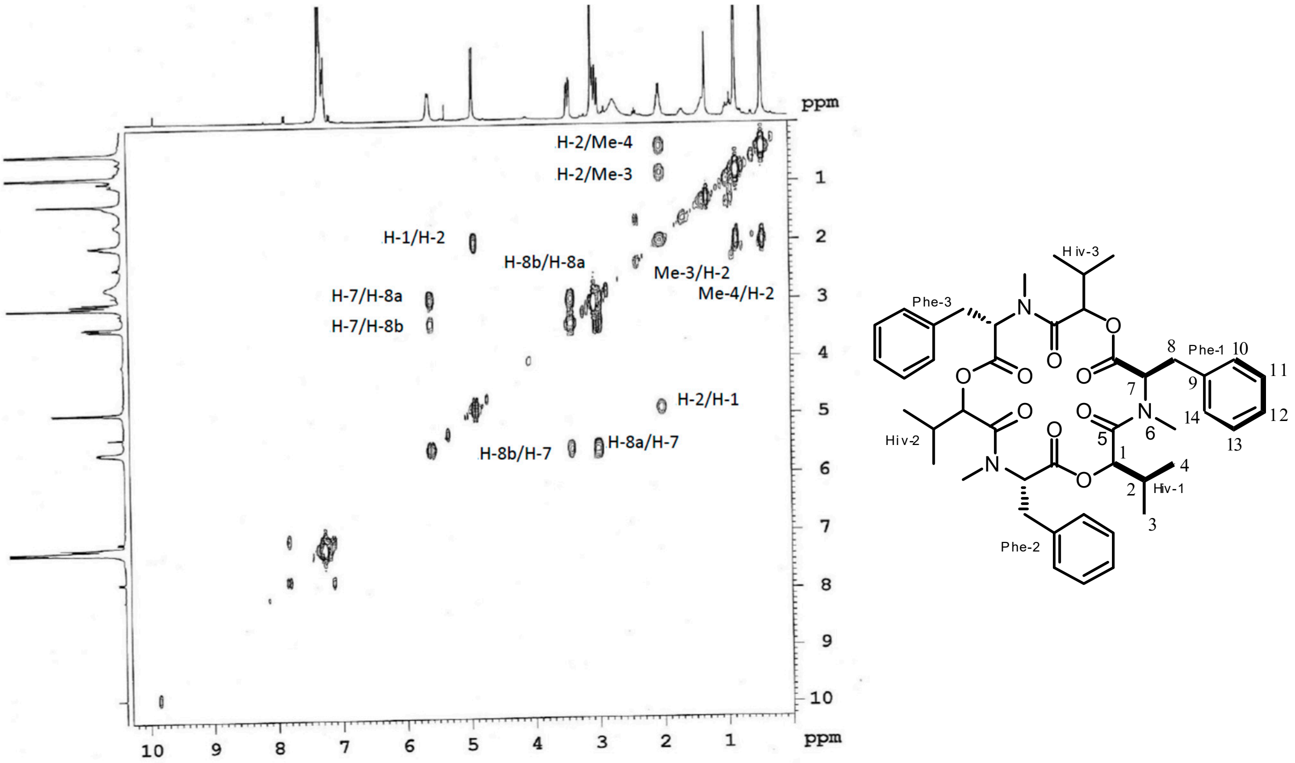

Figure S35. COSY spectrum of compound **5**.

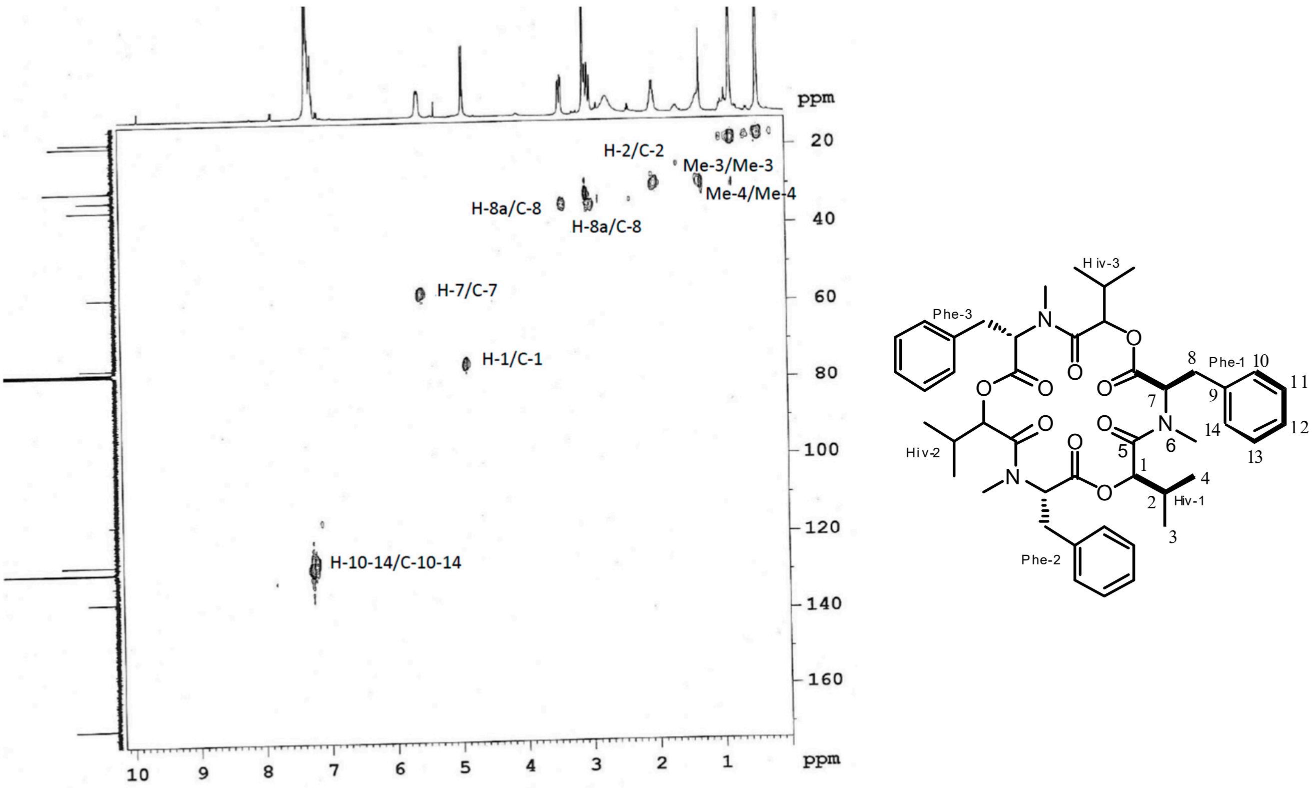

Figure S36. HSQC spectrum of compound **5.**

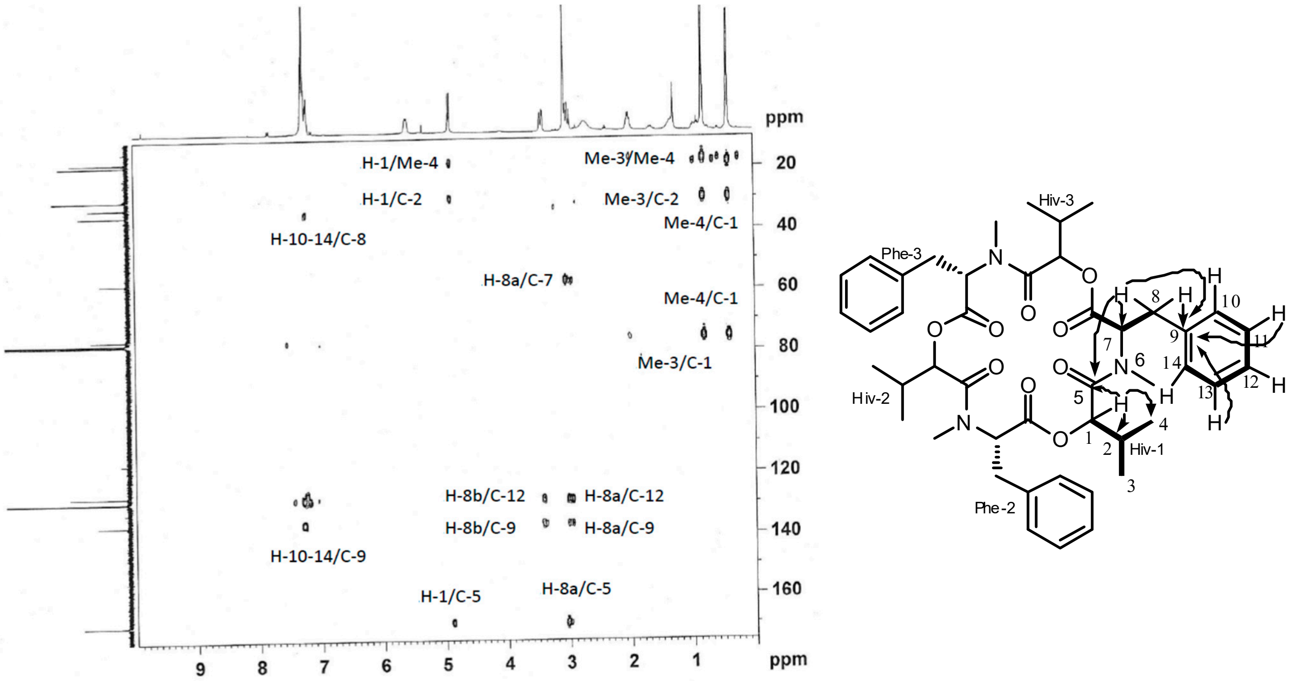

Figure S37. HMBC spectrum of compound **5.**

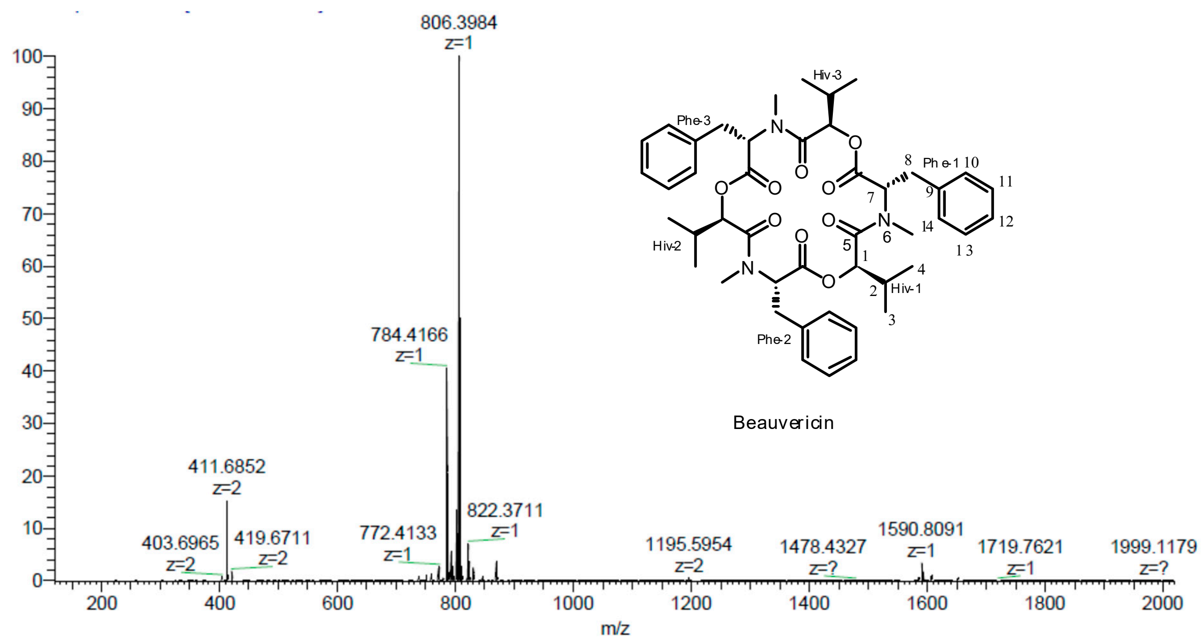

Figure S38. HRMS spectrum of compound **5.**

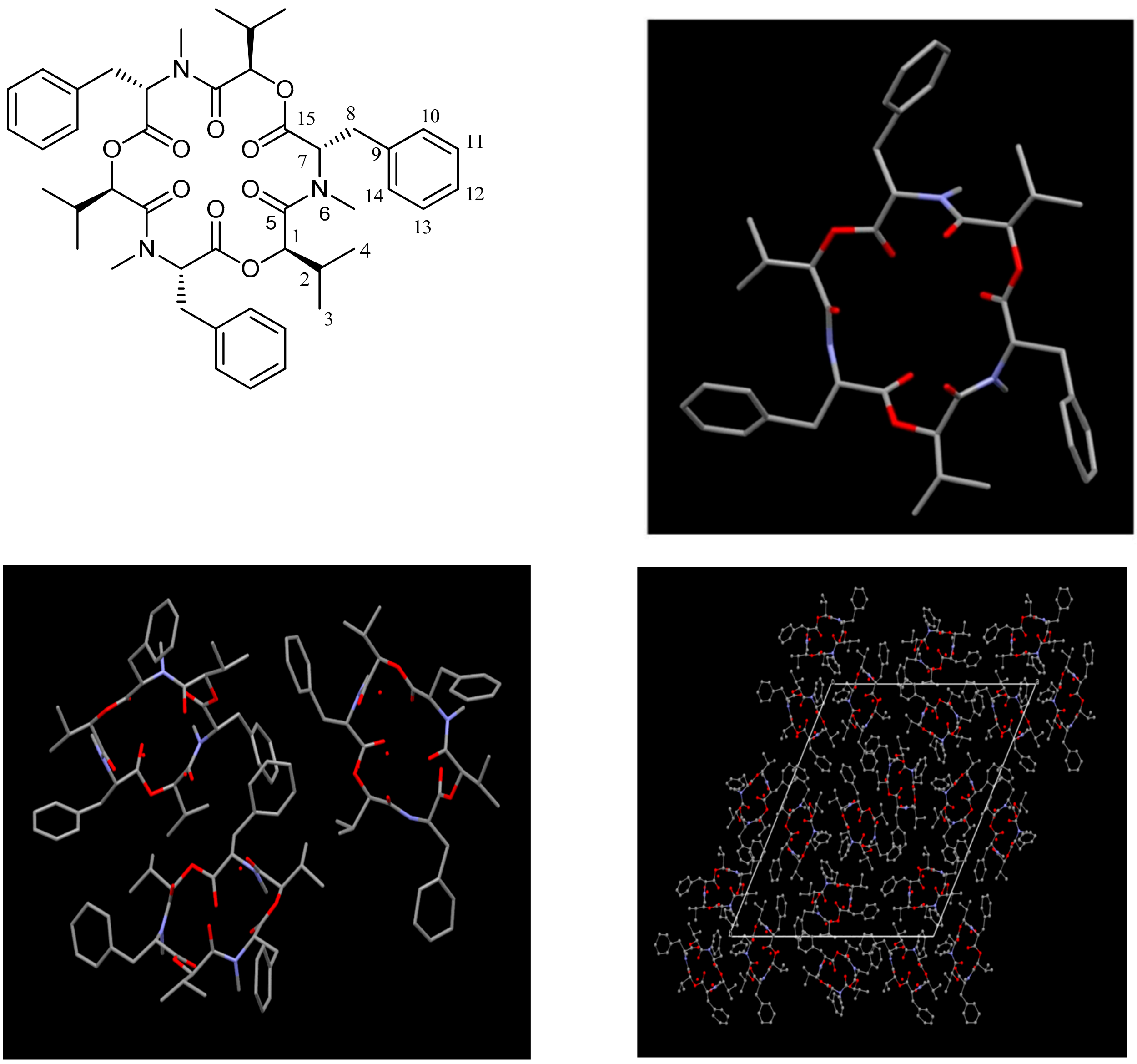

Figure S39. X-ray crystallography of compound **5.**

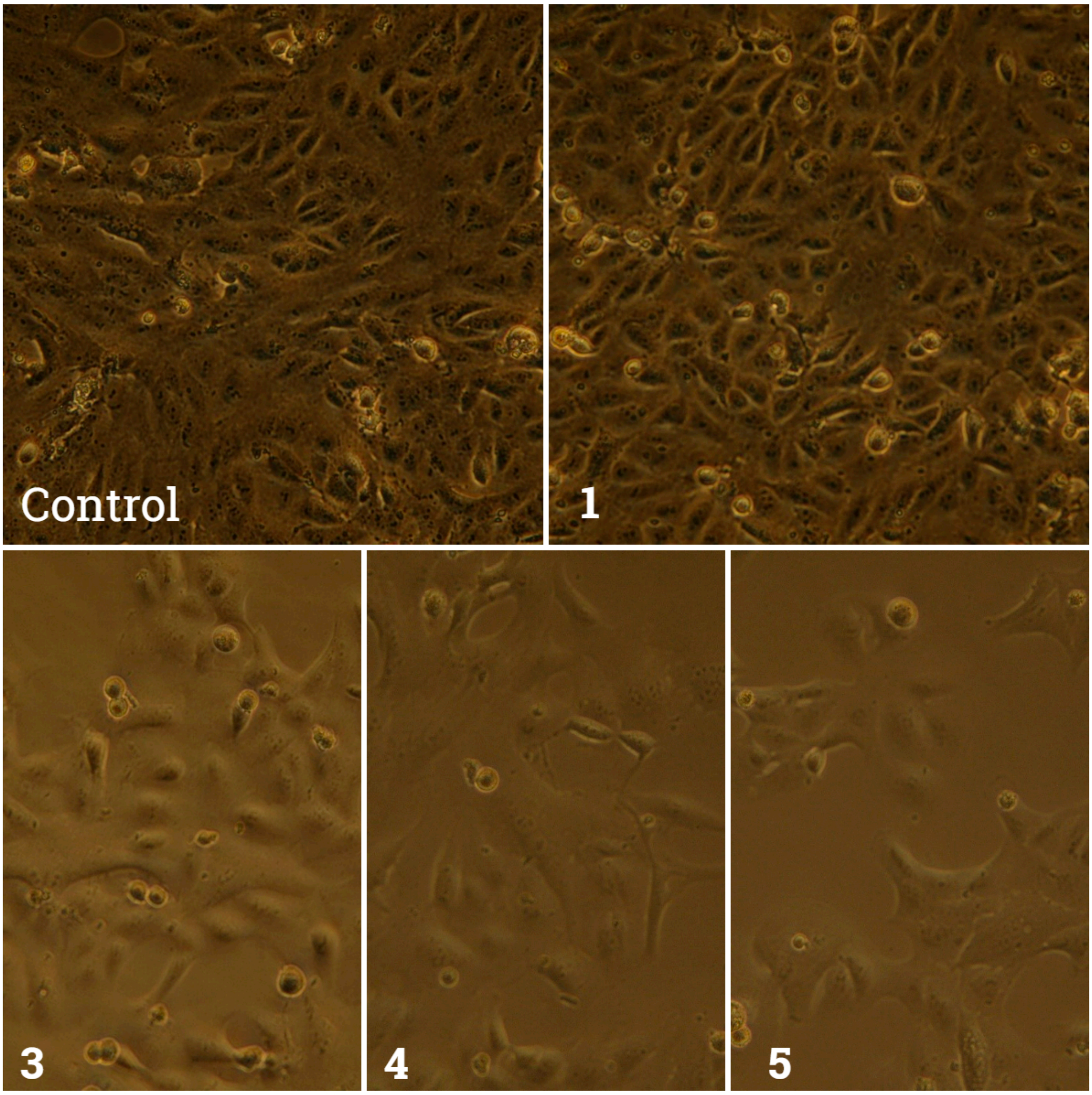

Figure S40. Effect of the compounds **1**, **3**, **4** and **5** on Vero cells compared to the control DMSO. All the compounds reduced the cell viability at the concentration of 20.0μM except compound **1**. Here, control = DMSO, **1** = 3*β*,5*α*-dihydroxy-ergosta-7,22-diene-6-one, **3** = *p*-hydroxybenzaldehyde, **4** = 3-(*R*)-7-butyl-6,8-dihydroxy-3-pent-11-enylisochroman-1-one, **5** = Beauvericin.

Compound 1

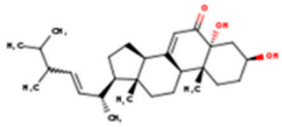

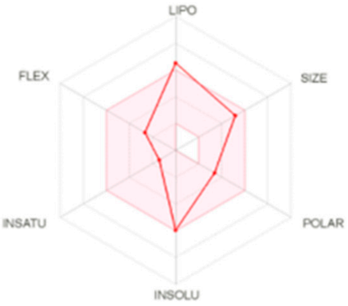

SMILES O[C@H]1CC[C@]2([C@@](C1)(O)C(=O)C=C1[C@@H]2CC[C@]2([C@H]1CC[C@@H]2[C@@H](C=CC(C(C)C)C)C)C)C

Physicochemical Properties	
Formula	C28H44O3
Molecular weight	428.65 g/mol
Num. heavy atoms	31
Num. arom. heavy atoms	0
Fraction Csp3	0.82
Num. rotatable bonds	4
Num. H-bond acceptors	3
Num. H-bond donors	2
Molar Refractivity	129.35
TPSA	57.53 Å²

Lipophilicity	
Log $P_{o/w}$ (iLOGP)	4.07
Log $P_{o/w}$ (XLOGP3)	5.93
Log $P_{o/w}$ (WLOGP)	5.70
Log $P_{o/w}$ (MLOGP)	4.52
Log $P_{o/w}$ (SILICOS-IT)	5.66
Consensus Log $P_{o/w}$	5.18

Water Solubility	
Log *S* (ESOL)	-5.97
Solubility	4.60e-04 mg/ml ; 1.07e-06 mol/l
Class	Moderately soluble
Log *S* (Ali)	-6.91
Solubility	5.24e-05 mg/ml ; 1.22e-07 mol/l
Class	Poorly soluble
Log *S* (SILICOS-IT)	-4.58
Solubility	1.12e-02 mg/ml ; 2.61e-05 mol/l
Class	Moderately soluble

Pharmacokinetics	
GI absorption	High
BBB permeant	No
P-gp substrate	No
CYP1A2 inhibitor	No
CYP2C19 inhibitor	No
CYP2C9 inhibitor	No
CYP2D6 inhibitor	No
CYP3A4 inhibitor	No
Log K_p (skin permeation)	-4.70 cm/s

Druglikeness	
Lipinski	Yes; 1 violation: MLOGP>4.15
Ghose	No; 2 violations: WLOGP>5.6, #atoms>70
Veber	Yes
Egan	Yes
Muegge	No; 1 violation: XLOGP3>5
Bioavailability Score	0.55

Medicinal Chemistry	
PAINS	0 alert
Brenk	1 alert: isolated_alkene
Leadlikeness	No; 2 violations: MW>350, XLOGP3>3.5
Synthetic accessibility	6.20

Figure S41. Outcomes of compound **1** while running in SwissADME.

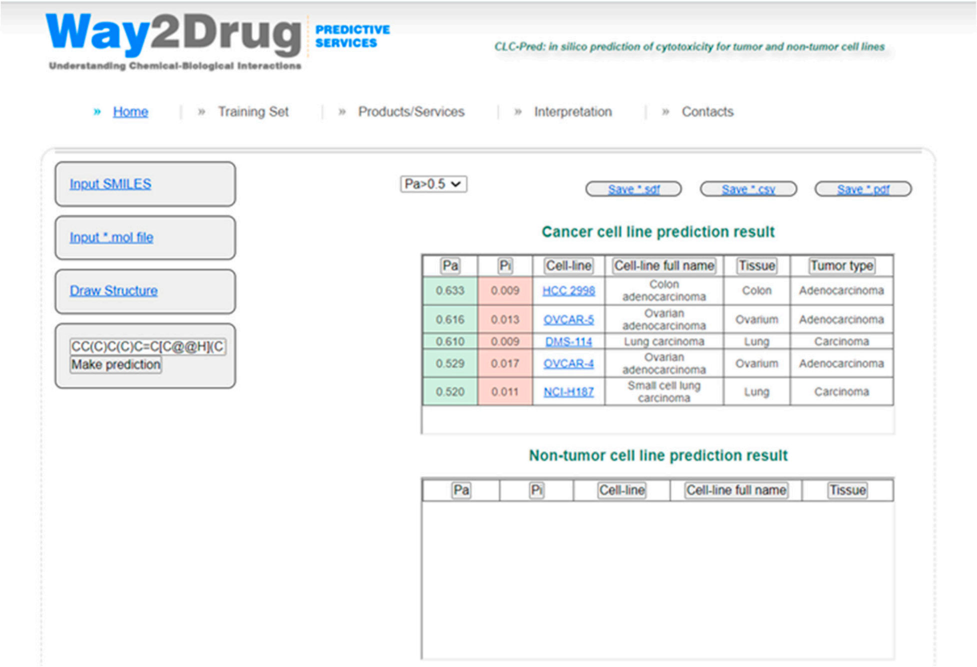

Figure S42. Outcomes of compound **1** while running in Cell Line Cytotoxicity Predictor (CLC-Pred) online tool.

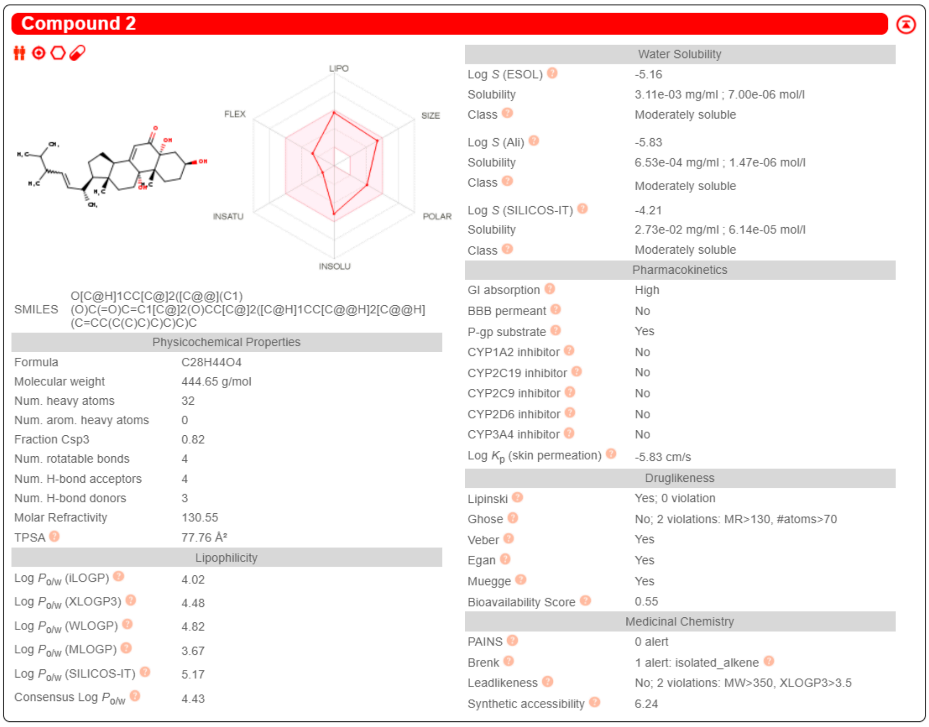

Figure S43. Outcomes of compound **2** while running in SwissADME.

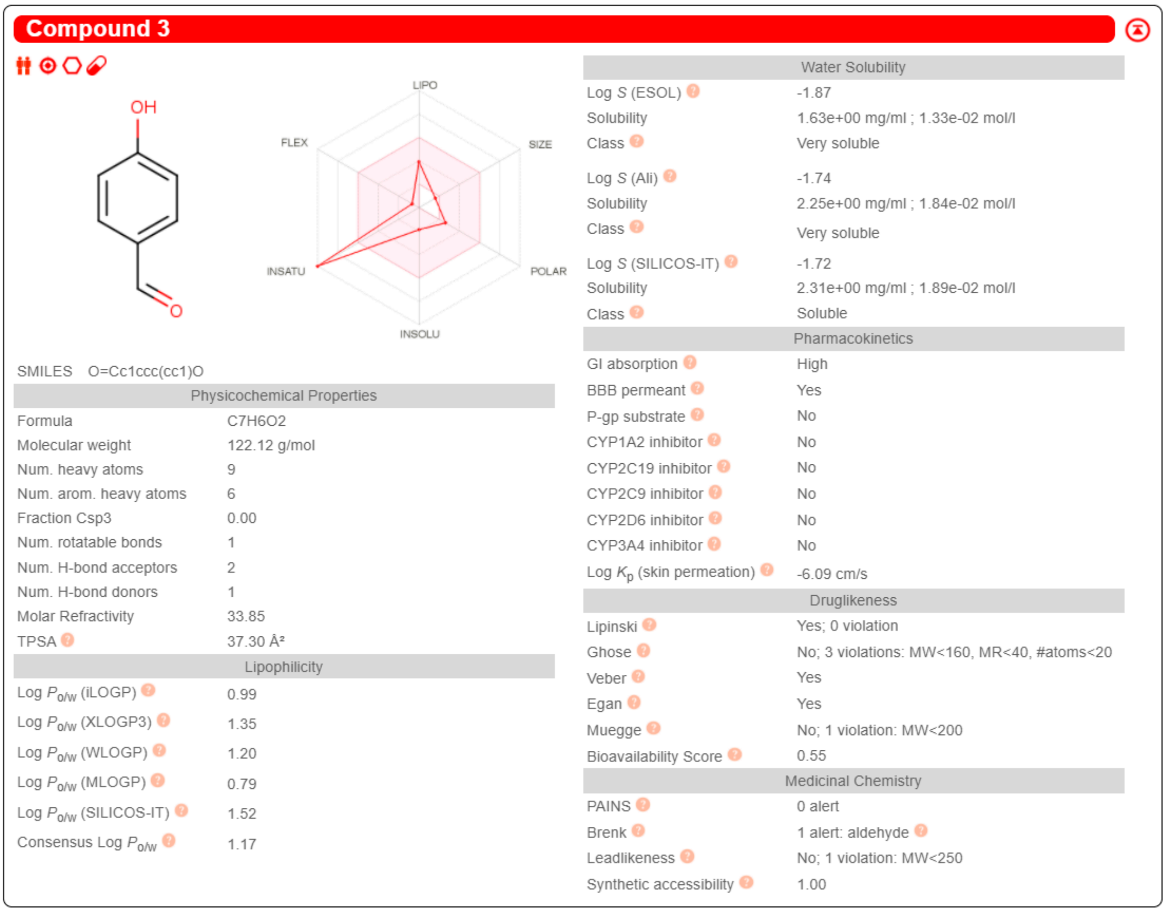

Figure S44. Outcomes of compound **3** while running in SwissADME.

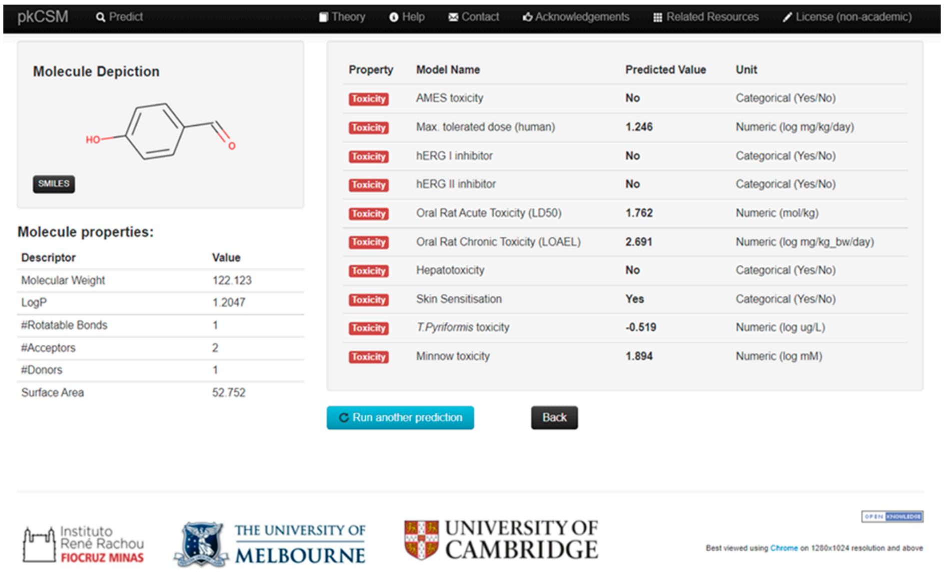

Figure S45. Outcomes of compound **3** while running in pkCSM online tool.

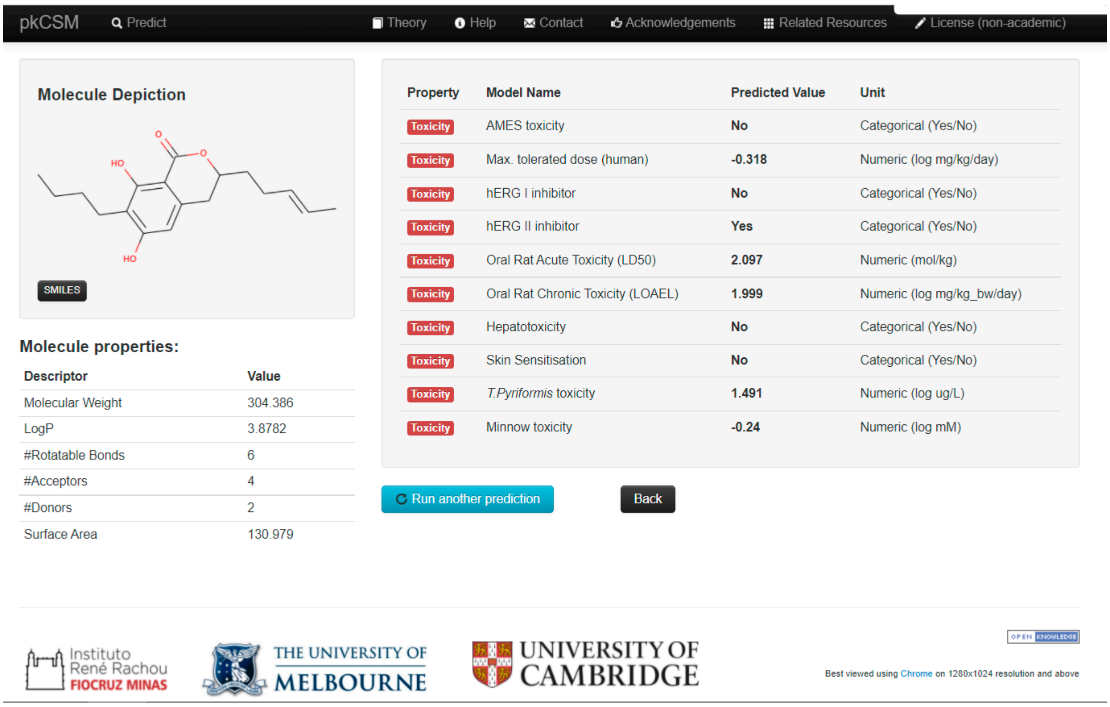

Figure S46. Outcomes of compound **4** while running in pkCSM online tool.

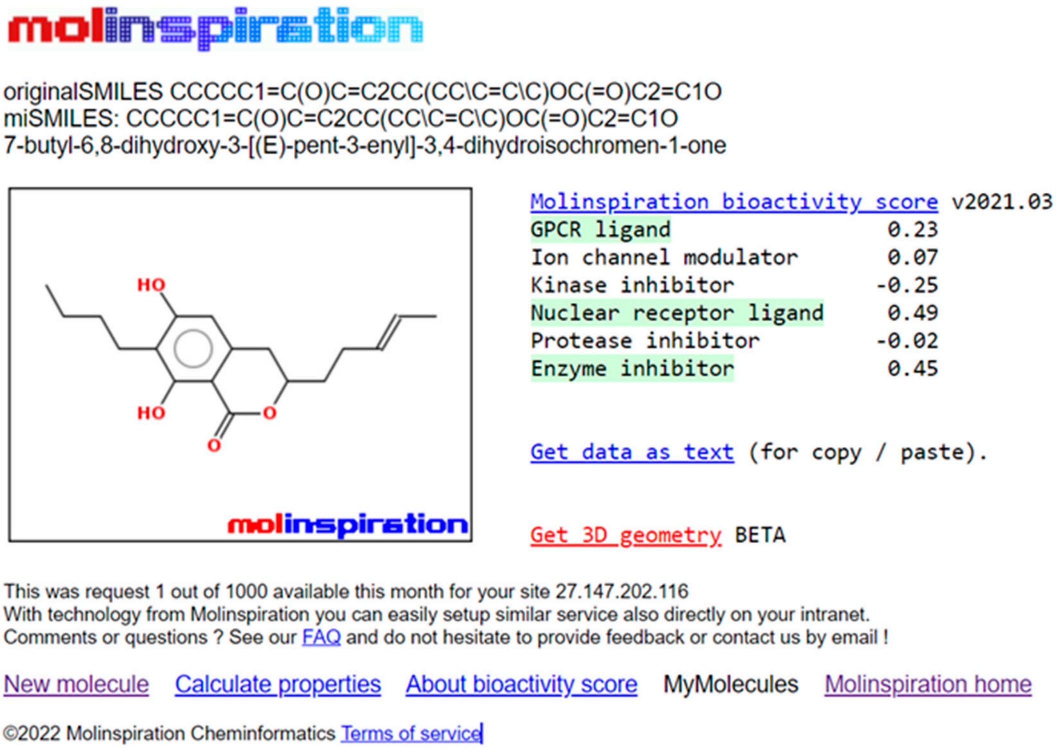

Figure S47. Outcomes of compound **4** while running in Molinspiration online tool.

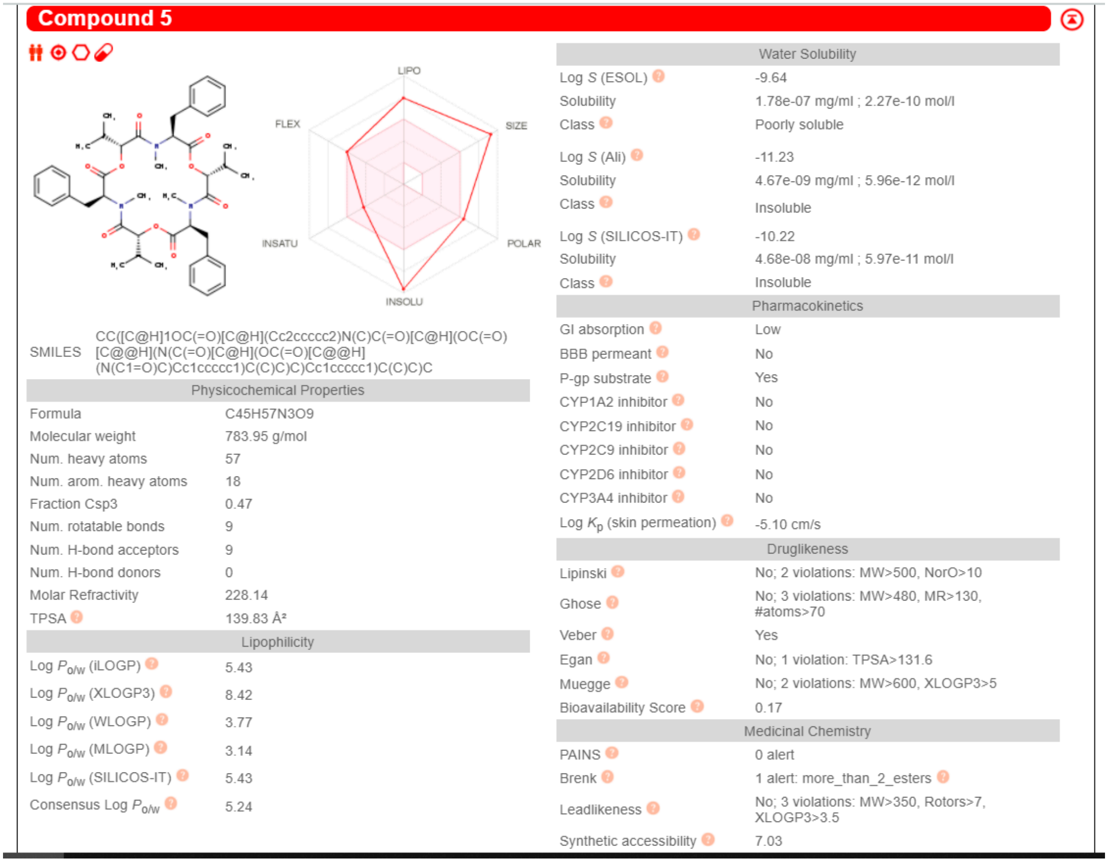

Figure S48. Outcomes of compound **5** while running in SwissADME.

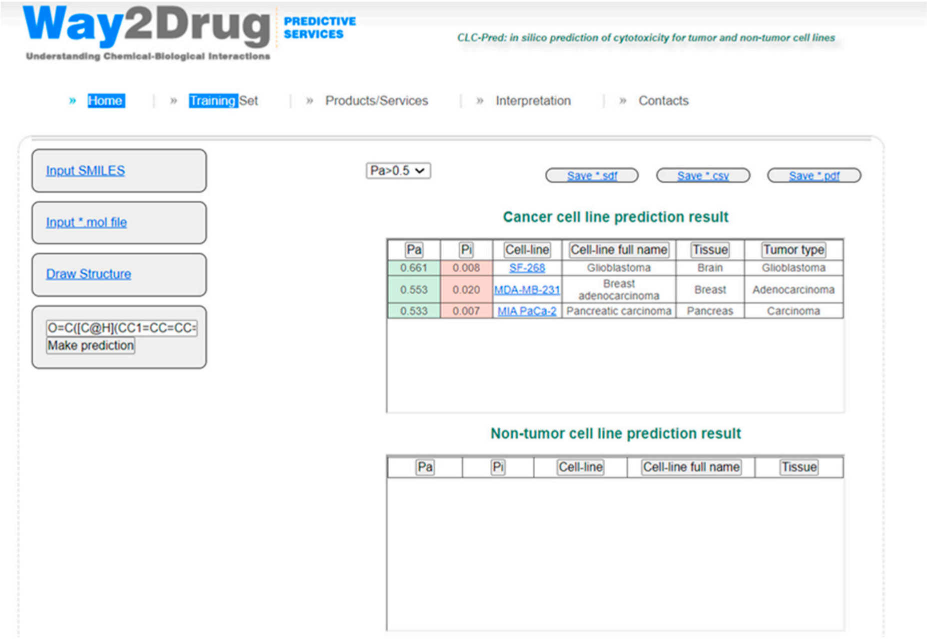

Figure S49. Outcomes of compound **5** while running in Cell Line Cytotoxicity Predictor (CLC-Pred) online tool.

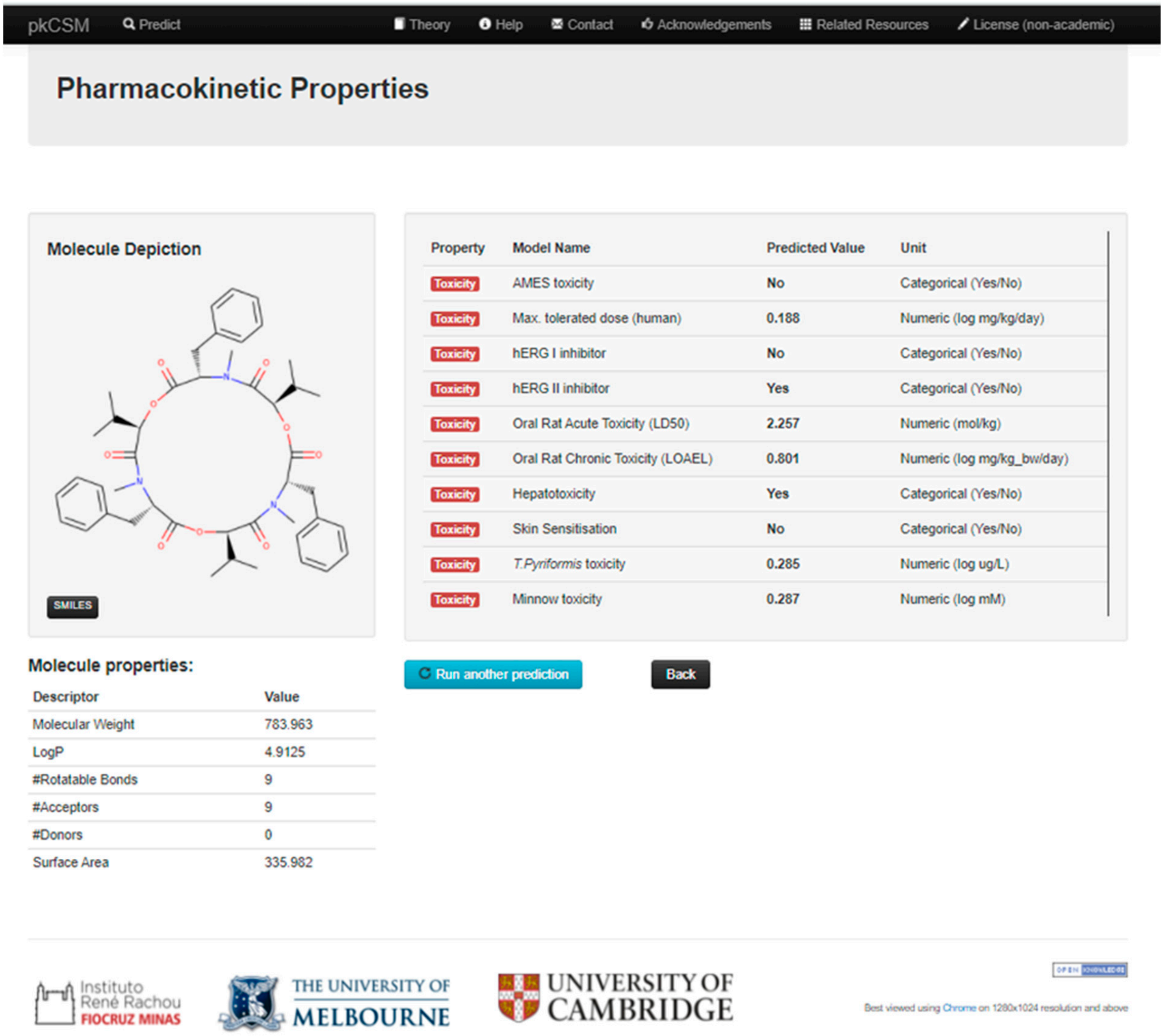

Figure S50. Outcomes of compound **5** while running in pkCSM online tool.

References

1. Rahman, M.A.; Uddin, S.B.; Wilcock, C.C. Indigenous knowledge of herbal medicine in Bangladesh-Treatment of jaundice by tribal communities of the Hill tract Districts. *Hamdard Medicus* **2003**, *64*, 25–28.
2. Rahmatullah, M.; Rani, S. Pk.; Imran, M.A.; Jahan, R. The Khasia tribe of Sylhet District, Bangladesh, and their fast-disappearing knowledge of medicinal plants. *J. Altern. Complement. Med.* **2013**, *19*, 599–606.
3. Nath, A.; Maiti, G.G. *Shuktani*- a new ethno-medico recipe among the Sylheti Bengali community of Barak valley, Southern Assam, India. *Indian, J. Tradit. Knowl*. **2012**, *11*, 156–160.
4. Roy, A.; Biswas, S.K.; Chowdhury, A.; Shill, M.C.; Raihan, S.Z.; Muhit, A. Phytochemical screening, cytotoxicity and antibacterial activities of two Bangladeshi medicinal plants. *Pak. J. Biol. Sci*. **2011**, *14*, 905–908.
